# A feature-centric decision-making framework for diagnosing and enhancing system efficiency in intelligent multi-agent manufacturing

**DOI:** 10.1038/s41598-026-56932-5

**Published:** 2026-06-09

**Authors:** Kusum Yadav, Lulwah M. Alkwai, Shahad Almansour, Debashis Dutta

**Affiliations:** 1 College of Computer Science and Engineering, University of Ha’il, Ha’il, Kingdom of Saudi Arabia; 2 Applied College, University of Hail, Ha’il, Kingdom of Saudi Arabia; 3 Sharda School of Engineering and Sciences, Sharda University, Greater Noida, UP India; 4 Department of computers Techniques engineering, College of technical engineering, The Islamic University, Najaf, Iraq

**Keywords:** Smart manufacturing, Multi-agent systems, Recursive feature elimination, ANOVA variance decomposition, SHAP explainability, Engineering, Mathematics and computing

## Abstract

This research offers a feature-centric hybrid predictive framework to forecast the efficiency of the system in intelligent multi-agent manufacturing environments. By combining operational, learning-based, and cyber-physical indicators, the suggested approach caters to the growing demand for interpretable, resilient, and high-performance analytics in Industry 4.0/5.0 contexts. The paper presents a structured pipeline that involves recursive feature elimination for a principled feature selection, ANOVA-based sensitivity assessment for statistical variance attribution, and SHAP-based global explainability for model-embedded interpretability. To boost the predictive accuracy, three tree-based baseline learners—decision trees, random forests, CatBoost, and extra trees—are combined with two recent meta-heuristic optimizers: prairie dog optimization (PDO) and electric eel foraging optimization. The experimental results indicate that the hybrids, especially the PDO-enhanced random forest and extra trees models, lead to a significant increase in accuracy, stability, and error reduction across all the test stages. Sensitivity analyses continuously point out production efficiency, machine usage, Q-value, and security event as the main predictors, which confirms the multi-modal nature of industrial performance dynamics. The results emphasize the viability of feature-driven modeling and biologically inspired optimization in producing robust and interpretable outcomes that are suitable for practical smart manufacturing applications. This research adds a novel, explainable, and deployable predictive intelligence paradigm for modern multi-agent industrial systems as its contribution.

## Introduction

The manufacturing sector is undergoing a rapid transformation driven by the convergence of artificial intelligence, Internet-of-Things (IoT) sensing^1^, and distributed control architectures. Smart factories of the future are heavily dependent on multi-agent control, reinforcement learning, and adaptive controllers (e.g., fuzzy-PID) to coordinate heterogeneous equipment, optimize throughput, and respond to dynamic disturbances. The move is largely driven by the need to meet business pressures more effectively: reduced downtime, higher quality yields, lower energy consumption, and increased resilience to cyber-physical disruptions^2,3^. Recent industry surveys demonstrate that organizations investing in smart manufacturing and advanced analytics are experiencing tangible benefits in agility and productivity, although they are also facing new operational and cybersecurity challenges that necessitate the use of more intelligent and explainable analytics^4–6^. According to the scientific view, predictive modeling in manufacturing has to satisfy several conflicting requirements simultaneously. The models should be (1) accurate in different operating regimes, (2) robust to noisy or adversarial inputs (e.g., security events), (3) computationally efficient for edge or near-edge deployment, and (4) interpretable to engineering stakeholders who need to trust and act on model outputs^7^. The smart manufacturing multi-agent control dataset is a perfect example of these requirements by the way it integrates agent-level signals (Q-values, task execution metrics), resource metrics (machine usage, energy consumption), control signals (fuzzy-PID adjustments), and security/anomaly logs^8–10^. The multi-modal nature of this composition is an excellent testbed for hybrid, feature-driven predictive strategies that can achieve both high performance and explainability^11,12^.

The motivators behind this research are, thus, two main points. Firstly, existing black-box methods (pure deep learning or monolithic ensembles) typically produce strong point forecasts but give very little insight as to why performance changes occur. This is a problem for the implementation of operations. Secondly, manufacturing operational datasets contain a combination of discrete event indicators and continuous, almost normal signals; therefore, obtaining reliable, low-dimensional feature representations that retain the operational semantics is the key to building models that can generalize across scenarios. Consequently, this study establishes a feature-centric hybrid modeling pipeline that uses principled selection (RFE), explainability (SHAP/ANOVA), and hybrid ensemble structures to a great extent both predictive accuracy and practical interpretability, thus meeting the industrial needs for trustworthy, deployable predictive intelligence, which is of an urgent nature.

Recent studies have shown that, within the scope of hybrid machine learning applications in manufacturing systems, the integration of multiple learning paradigms significantly enhances prediction reliability and process understanding, thereby clearly establishing the position of the adopted approach within the existing literature^13–16^. Recent studies have shown that, within the scope of industrial sensor-based predictive modeling, the effective use of multi-source data is essential for capturing complex system dynamics and ensuring reliable prediction performance^17^.

Research devoted to predictive maintenance, energy forecasting, and quality prediction within smart manufacturing has dramatically increased over the last two years. Reviews of such research highlight that AI-driven predictive maintenance pipelines that involve IoT sensing, streaming analytics, and supervised learning can significantly reduce downtime if they are domain knowledge-integrated. However, these reviews also note that such systems rarely consider interpretability and adversarial resilience^18^. Recent work is furthering hybridization (classical ML + specialized optimizers) to enhance stability and lower overfitting in small/medium industrial datasets. As a result, hybrid regression/ensemble methods and explainability-aware pruning have been found to both raise performance and stakeholder trust^19^.

Feature selection is still at the core of most discussions: one can still use greedy and wrapper methods such as recursive feature elimination (RFE)^20^, and the recent variants of RFE aim not only to automate the process of elimination but also to reduce the bias by integrating representative sampling or stacking strategies. These investigations acknowledge RFE as a tool for industrial datasets of high dimensionality but of moderate size, while they also reveal drawbacks—RFE may be a time-consuming process and can be very dependent on the choice of a base estimator, which can in turn direct the retained subset^21^.

Recent advances in intelligent manufacturing and hybrid AI systems highlight the increasing role of optimization-enhanced machine learning and explainable decision frameworks. Studies such as those reported in recent hybrid AI architectures^22^ demonstrate the effectiveness of combining data-driven models with system-level analytics for industrial decision support. Similarly, optimization-driven predictive frameworks^23^ emphasize the importance of integrating metaheuristic strategies with machine learning models to enhance prediction accuracy and robustness.

Cognitive and agent-based intelligent systems^24^ further extend this direction by incorporating adaptive decision-making mechanisms in multi-agent environments. More recent developments in optimization-enhanced machine learning^25^ reinforce the effectiveness of hybrid learning strategies in complex industrial scenarios. In addition, emerging studies in feature representation learning and variability-aware AI models^26–28^ highlight the importance of robust feature engineering and adaptive model design.

Despite these advancements, most existing approaches either prioritize predictive performance or model complexity, often lacking a unified feature-centric framework that integrates feature selection, statistical sensitivity analysis, optimization, and explainability within a single coherent pipeline. This gap motivates the proposed study.

Explainable AI (XAI) has also developed as a commercially viable subfield in production. Research studies that compare SHAP, LIME, PDP, and similar methods reveal that global and local explanations bring real and significant advantages in defect detection, energy forecasting, and maintenance planning^29^. Nevertheless, the comparative research indicates that XAI instruments vary greatly in their steadiness and in their concordance with domain knowledge; thus, the issue of deploying them mindlessly in operations without checking against physics or domain constraints arises^30^. Despite advances, several persistent limitations remain across the literature:

1. Tradeoff between interpretability and accuracy: many high-accuracy models are black boxes; interpretable models can underperform unless carefully engineered.
2. Insufficient robustness to security events: most predictive frameworks ignore adversarial or anomalous event streams that materially change system behavior.
3. Limited benchmarks for multi-agent control settings: few public studies evaluate models on datasets that simultaneously include agent learning signals (Q-values), control adjustments, and security logs.
4. Computational cost and deployment gap: advanced feature selection and ensemble tuning are often expensive, hindering edge deployment.

To summarize the recent literature and highlight gaps, Table 1 presents a concise, novel synthesis of representative 2024–2025 studies, their methods, domains, and principal limitations.

**Table 1:** Representative recent works (2024–2025) and key limitations.

Year	Study	Method	Domain	Key Limitation
2024	AI-driven predictive maintenance^18^	Survey of ML/IoT	Predictive maintenance, smart manufacturing	Emphasizes methods but is limited to XAI deployment
2024	Explainable AI for predictive maintenance^19^	XAI + ML pipelines	Maintenance forecasting	XAI stability is inconsistent across methods
2024	RFE automation study^21^	Automated RFE variants	Feature selection	Computational cost; base-estimator bias
2024	Hybrid ML models^29^	Hybrid ensembles	Energy/consumption forecasting	Limited evaluation on multi-agent datasets
2025	Comparative XAI methods^30^	SHAP/LIME/PDP comparisons	Defect prediction	Divergence among XAI outputs; domain alignment needed

Unlike existing hybrid machine learning pipelines that primarily focus on predictive accuracy, the proposed framework introduces a structured feature-centric intelligence architecture that integrates feature selection, statistical sensitivity analysis, metaheuristic optimization, and explainable AI into a unified industrial decision-support pipeline. The major aim of this research is to establish a highly accurate, feature-focused, and explainable predictive model that can efficiently simulate system performance in intelligent multi-agent manufacturing environments. Because of the extensive use of autonomous agents, IoT sensors, and cyber-physical monitoring tools, the authors of the paper intend to surpass the weaknesses of the present black-box methods which have high predictive power but lack interpretability, resilience, and operational trustworthiness. The goal is not just to get the best results but also to provide clear insight into the underlying causes of system behavior—a core industrial decision-making requirement.

The study brings in a few new aspects in analytics for smart manufacturing, which can be considered as novel elements. To begin with, it presents a thoroughly planned feature-engineering pipeline revolving around recursive feature elimination (RFE) that facilitates the principled picking out of the most influential operational, learning-based, and security-sensitive variables. Secondly, the combined dual-perspective sensitivity analysis—ANOVA for variance-based statistical decomposition and SHAP for model-aware feature attribution—integrated into the system creates a comprehensive interpretability foundation that is very rarely considered jointly in industrial research. Thirdly, the study elaborated on the reasons and advantages of six hybrid predictive models constructed by the integration of three potent tree-based learners and two recent meta-heuristic optimization algorithms: prairie dog optimization (PDO) and Electric Eel Foraging Optimization (EEFO). These optimizers bring about the changes in hyperparameters by the biologically inspired exploration–exploitation mechanisms and thus, laudably improve the model’s generalization and convergence.

Empirical results emphasize that the hybrid models substantially outperform the baseline learners and thereby provide evidence for the interaction between optimized hyperparameters and high-value features like production efficiency, machine usage, Q-value, and security event. By integrating interpretability, sensitivity recognition, and excellent predictive accuracy, the proposed framework is a practical, reliable, and expandable solution for future intelligent manufacturing systems.

## Data and feature engineering pipeline

### Dataset description and statistical characteristics

The data set for this paper is the smart manufacturing multi-agent control dataset, a thorough benchmark for evaluating intelligent, decentralized manufacturing systems. The dataset models a multi-agent industrial scenario where four types of autonomous agents can perform tasks, change machine behavior, and respond to changing production conditions by reinforcement learning methods and fuzzy PID control techniques^31,32^. In doing so, it offers an extremely rich and very realistic depiction of sophisticated Industry 4.0/5.0-future scenarios. Besides operational data, the dataset has also been instrumented with security event logs, anomaly diagnostics, and control system disturbances, allowing researchers to extend their work into cybersecurity, optimization, process control, and smart automation domains. The target variable, system efficiency, denotes the process performance and robustness of the manufacturing system, hence, combining a multiplicity of operational and cyber-physical factors. The variables in the dataset are divided into two primary distribution categories. Discontinuously distributed uniformly random discrete indicators, such as security event (0–3) and anomaly detected (0–1), are those which show abrupt system state transitions and cyber-physical disturbances. These features serve as high-value trigger signals for pinpointing deviations in the operational scene. The rest of the variables are continuous and roughly normally distributed, and they are able to capture the subtle control and production behavior changes. As an example, execution time shows significant operational variability (mean = 12.5169, SD = 4.3406), whereas Q-Value is very narrowly spread around 0.7493, thus indicating a stable agent learning process.

Essential operational metrics, machine usage, energy consumption, and production efficiency, are characterized by wider ranges that depict genuine manufacturing variations. System Efficiency, with a mean of 62.2431, serves as a composite performance output that integrates the effects of these different dimensions. Table 2 outlines the distribution type and statistical properties of the variables, which represent the foundational input space for feature engineering later on.

**Table 2:** Input variables consideration.

Distribution	Variable	Indicator			
		Upper bound		Lower bound	
Uniform	Security event	3		0	
	A1maly detected	1		0	

		Max.	Min.	Avg.	St. Dev.
Normal	Execution time	20.0000	5.0001	12.5169	4.3406
	Q value	1.0000	0.5000	0.7493	0.1445
	Machine usage	99.9997	70.0006	85.0110	8.6630
	Energy consumption	59.9994	30.0004	44.9901	8.6508
	Production efficiency	100.0000	80.0000	90.0382	5.7651
	System efficiency	76.0904	43.8515	62.2431	4.7988

### Exploratory data analysis (EDA) and feature distributions

**Fig. 1:**
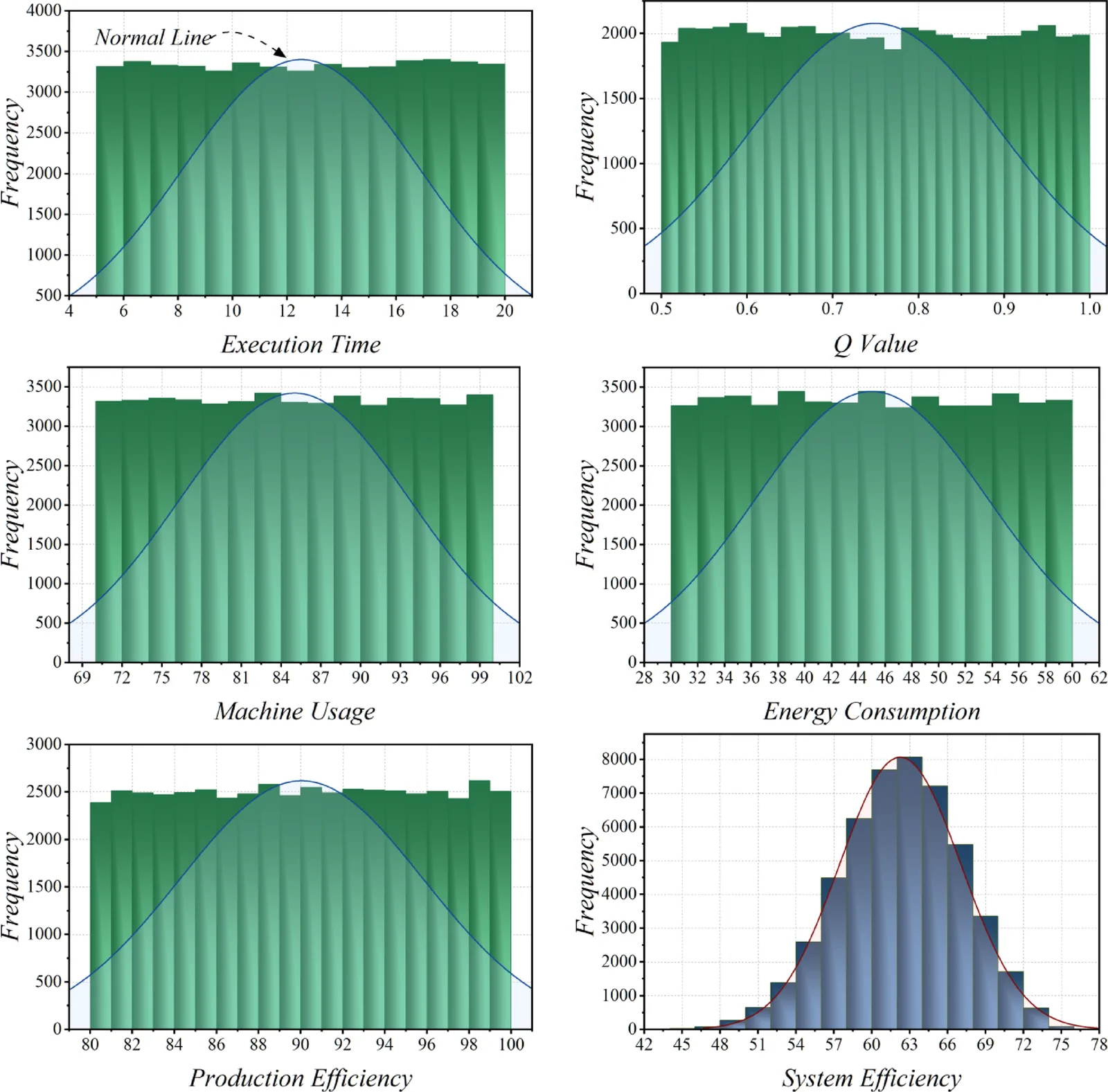
Histogram plot depicting the statistical distribution of the dataset variables.

The exploratory analysis phase is mainly about discovering the structural patterns, variability, and distribution characteristics that will be used to guide the subsequent learning process. All the variables in Fig. 1 are represented by their histogram plots, which vividly demonstrate the statistical diversity of the dataset. The uniform features reveal sharp, discrete segmentations that correspond to event-driven system states, thus confirming their function as categorical performance and security indicators. On the other hand, the continuous features have smooth, bell-shaped distributions which are indicative of near-normal behavior. These distributional patterns serve as a confirmation that variance-based feature assessment, normalization, and hybrid model integration are appropriate. The histograms uncover quite a few operational insights that are worth noticing:

- Execution time and energy consumption are good examples of natural workload fluctuations that have been driven by environmental and agent-level decision variability.
- Production efficiency and machine usage are closely packed around the high-performance zones, which is in line with stable industrial throughput.
- System Efficiency has a wider range of values, as it is very sensitive to both operational quality and security disturbances.

These discoveries are the basis for the development of the feature engineering pipeline, which is instrumental in scaling, sensitivity analysis, and hybrid model construction decisions.

### Feature selection strategy using recursive feature elimination (RFE)

**Fig. 2:**
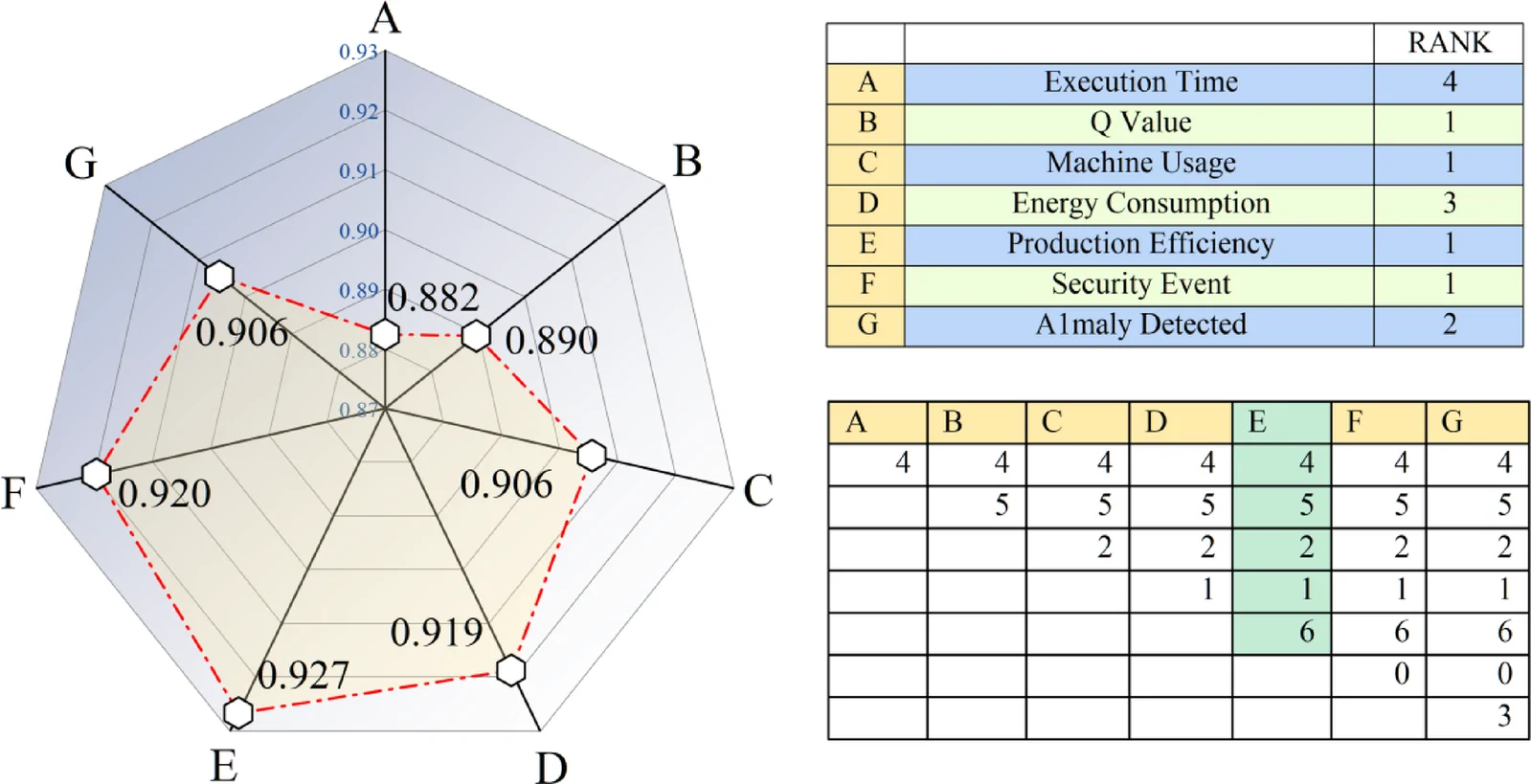
Feature selection results obtained using the RFE method.

An RFE strategy was employed to single out the most influential predictors for system performance modeling. RFE^20^ judges the contribution of each input variable iteratively by training a base estimator, a tree-based learner in this case, and gradually removing the features that are least informative until the optimal subset is found. The result of this operation is a ranking that is more robustly tied to the prediction power than to the statistical association. The RFE outcomes, shown in Fig. 2, signify the presence of a very distinct hierarchy of the input concepts.

Production efficiency, machine usage, Q value, and security event rank thus represent the most significant predictors that can be explained by their direct impact on the operational dynamics area and on the resilience of the system. Features that have a moderate impact are energy consumption and anomaly detected, both providing essential contextual information related to resource utilization and cyber-physical disturbances. Execution Time, which is a relatively low rank factor, is given the somewhat lower position in the multivariate predictive landscape because of its less dominant influence.

The radar chart in Fig. 2 shows the importance scores that have been normalized for all the variables, while the ranking matrices that go along with them provide a structured comparison of the elimination sequence over several RFE iterations. When taken together, these revelations confirm that the efficiency of the system is mostly controlled by the triple of attributes related to productivity, utilization, and stability that have been duly prioritized for the next hybrid modeling pipeline.

### Pre-processing, normalization, and dataset partitioning

To maintain data integrity, comparability among features, and the best performance of learning algorithms, a pre-processing pipeline with a structured approach was utilized. The pipeline included three major steps: data cleansing, normalization, and stratified dataset partitioning. Initially, the dataset was scrutinized for missing, inconsistent, or anomalous entries. Since the data had a synthetic multi-agent nature, there were no missing values; however, the outlier inspection was carried out to confirm the stability of the continuous variables, such as execution time, machine usage, and energy consumption. All the values were within the expected operational ranges defined by the manufacturing process; thus, they can be used directly in downstream operations without the need for imputation.

In order to avoid the learning algorithms’ scale-induced bias, the normalization of all continuous variables was done by min–max scaling. This method keeps the distributional characteristics that were identified during the exploratory analysis; at the same time, it ensures that each feature makes a proportional contribution to the learning process. The uniform discrete variables (security event and anomaly detected) were kept in their original categorical form because normalization would have changed their encoded event-level semantics.

Lastly, the dataset was divided into training, validation, and testing subsets by means of stratified sampling to keep the same distributions of continuous metrics and binary security indicators. The split chosen allowed for unbiased performance evaluation and, at the same time, robust hyperparameter tuning in the subsequent stages of the modeling pipeline. Such a structured partitioning is crucial for the hybrid models to be able to generalize effectively under different operational and security conditions.

### Reproducibility and implementation details

All experiments were executed on a workstation equipped with an Intel Core i7 processor, 32 GB RAM, and NVIDIA RTX-series GPU acceleration. Model implementation and analysis were performed using Python-based scientific computing libraries. To facilitate reproducibility, the preprocessing configuration, optimization settings, feature selection strategy, and evaluation metrics have been explicitly documented in the manuscript. The dataset utilized in this study is publicly accessible/generated from benchmark industrial simulation sources. Implementation code and experimental configurations can be made available upon reasonable request (Table 3).

**Table 3:** Experimental and optimization settings.

Parameter	Value
Training split	80%
Validation split	10%
Testing split	10%
random seed	42
Normalization	Min–Max
PDO population size	30
EEFO population size	30
Optimization iterations	200
Programming language	Python 3.11
Frameworks	Scikit-learn, SHAP, NumPy

## Methodological framework

The research employs a structured methodological framework that combines (1) robust tree-based baseline learners, (2) hybrid meta-heuristic optimization of their hyperparameters, and (3) a feature-driven learning pipeline. With this setup, the models can identify nonlinear interactions, improve generalization, and keep interpretability, which are very important features in multi-agent smart manufacturing systems that use control, security, and operational signals.

### Baseline algorithms: RF, DT, and ET learning models

To this day, tree-based machine learning algorithms are the go-to solutions for industrial predictive modeling due to their interpretability, robustness, and nonlinear relationship representation capacity without the need for extensive pre-processing. The study has implemented four baseline models: decision trees (DT), random forests (RF), extra trees (ETR), and CatBoost regression (CATR).

#### Decision tree regressor (DTR)

A DTR divides the input space step by step by choosing the feature and split threshold that reduces impurities the most^33,34^. In the case of a regression task, the impurity measure most often used is variance reduction which is given as:

1 $$\Delta I=I\left(S\right)-\left(\frac{\left|{S}_{L}\right|}{\left|S\right|}I\left({S}_{L}\right)+\frac{\left|{S}_{R}\right|}{\left|S\right|}I\left({S}_{R}\right)\right)$$

*S*, ${S}_{L}$, and ${S}_{R}$ represent the parent, left-child, and right-child subsets.

The impurity is defined as:

2 $$I\left(S\right)=\frac{1}{\left|S\right|}\sum_{i\in S}{(yi-\overline{y })}^{2}$$

The final prediction for a sample is the mean value of the instances falling within the terminal leaf node.

#### Random forest regressor (RFR)

RFR algorithm alleviates the problem of overfitting by generating a set of random trees, which are trained with both bootstrap aggregation (bagging) and feature subsampling^35,36^. The output that corresponds to the majority (average) of all trees is the final prediction:

3 $$\widehat{y}=\frac{1}{T}\sum_{t=1}^{T}ft(x)$$

*T* is the number of decision trees, $ft\left(x\right)$ is the prediction of the *t*th tree.

Each tree is built by using a bootstrap sample of the training data set and a randomly selected subset of features at each split during training, which is a mixture that increases the differences between trees and, thus, greatly improves generalization.

#### Extra trees regressor (ETR)

The ETR algorithm goes beyond RF by adding maximum randomness in the split generation process^37^, where splits are chosen completely at random instead of by impurity reduction, and trees are trained on the entire dataset without bootstrap sampling, thus resulting in the following split rule:

4 $$\theta =\left(j,sj\right) \; with \; sj \sim U\left(\min \left(xj\right),\max \left(xj\right)\right)$$

*j* is a randomly selected feature index, ${s}_{j}$ is a random threshold sampled uniformly.

The prediction is obtained using the ensemble average:

5 $$\widehat{y}=\frac{1}{T}\sum_{t=1}^{T}{f}_{t}^{ET}(x)$$

As a result of its high randomness, ET gets lower variance, faster training, and stronger robustness to small fluctuations in industrial datasets.

#### CatBoost regression (CATR)

The CATR `method based on symmetric decision trees and ordered boosting, specifically designed to handle categorical features efficiently while reducing prediction bias^38^. At each iteration, a new decision tree is constructed to minimize the loss function by fitting the residual errors of the previous ensemble. The prediction model is expressed as:

6 $${\widehat{y}}_{i}=\sum_{t=1}^{T}\eta {f}_{t}\left({x}_{i}\right)$$

Here, *T* is the number of boosting iterations, *η* is the learning rate, ${f}_{t}({x}_{i})$ is the prediction of the *t*-th tree. The optimization process minimizes the regularized objective function defined as:

7 $$L=\sum_{i=1}^{N}l\left({y}_{i},{\widehat{y}}_{i}\right)+\sum_{t=1}^{T}\Omega \left({f}_{t}\right)$$

$l({y}_{i},{\widehat{y}}_{i})$ is the loss function, $\Omega ({f}_{t})$ represents the regularization term controlling tree complexity. CATR further employs ordered boosting to prevent target leakage during training and uses symmetric tree structures that improve computational efficiency and prediction consistency. Owing to these mechanisms, CATR demonstrates high robustness, fast convergence, and strong predictive performance for nonlinear and complex industrial regression datasets.

### Development of hybrid feature-driven models

Although baseline tree learners can yield powerful outcomes, their effectiveness depends on the careful choice of hyperparameters (e.g., number of estimators, depth, minimum samples per leaf). Hence, to enhance the precision and robustness of the models, the present work combines meta-heuristic optimizers with the baseline models, resulting in six hybrids:

**Figure:**
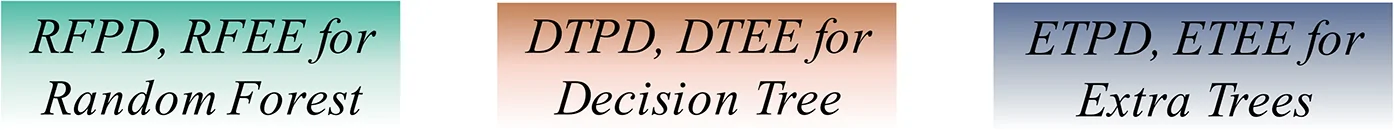

This work utilizes two novel optimization frameworks, EEFO and PDO, which were mainly used due to their high exploration capability, fast convergence, good performance in highly nonlinear search spaces, and stability on medium-sized industrial datasets.

#### Electric eel foraging optimization (EEFO)

EEFO^39^ mimics the hunting behavior of electric eels that emit electrical discharges, perform a spiral search, and attack with a pulse to find the most suitable prey. In the same way, the algorithm is switching between exploration (a broad search) and exploitation (a local search)^40^.

- Position update (Exploration)

8 $${X}_{i}^{t+1}={X}_{i}^{t}+{r}_{1}*\left({X}_{best}^{t}-{X}_{i}^{t}\right)+{r}_{2}*\mathrm{sin}\left(2\pi {r}_{3}\right)$$

${X}_{i}^{t}$ = position of the *i*-th candidate at iteration *t*, ${X}_{best}^{t}$ = globally best solution so far, ${r}_{1}, {r}_{2}, {r}_{3}$ = random values $\in [\mathrm{0,1}]$

- Electric discharge attack (Exploitation)

9 $${X}_{i}^{t+1}={X}_{best}^{t}+a*{e}^{-\beta di}*\left({X}_{j}^{t}-{X}_{k}^{t}\right)$$

*α*, *β* are control parameters, *di* is distance to prey (fitness gradient), ${X}_{j}^{t},{X}_{k}^{t}$ are two randomly selected solutions.

This formulation enables sharp local refinement around strong candidate regions.

- Fitness function

The target of minimization for regression is:

10 $$Fitness= RMSE= \sqrt{\frac{1}{n}\sum_{i=1}^{n}{({y}_{i}-{\widehat{y}}_{i})}^{2}}$$

EEFO searches for the hyperparameter set minimizing the RMSE.

#### Prairie dog optimization (PDO)

PDO^41^ represents the intricate social interaction of prairie dogs that includes activities like digging, watching, helping the group, and increasing the size of the burrow^42^. These conduct of the animals is converted into formalized exploration–exploitation cycles.

- Exploration phase (burrow scouting)

11 $${X}_{i}^{t+1}={X}_{i}^{t}+\gamma 1*\left|{X}_{i}^{t}-{X}_{rand}^{t}\right|*\epsilon$$

${X}_{rand}^{t}$is a random prairie dog (random solution),*γ 1*controls exploration strength,*ε*is a small random perturbation.

- Exploitation phase (cooperative digging)

12 $${X}_{i}^{t+1}={X}_{best}^{t}+ \gamma 2* \frac{{X}_{j}^{t}-{X}_{k}^{t}}{2}$$

*γ 2* is the exploitation coefficient, ${X}_{j}^{t}, {X}_{k}^{t}$ are random members of the colony.

- Social interaction adjustment

13 $${X}_{i}^{t+1}={X}_{i}^{t}+r*\left({X}_{best}^{t}-{X}_{i}^{t}\right)+\delta$$

*δ* is a correction factor intended to maintain diversity and prevent premature convergence.

#### Hybridization protocol

Each hybrid model uses the optimizer to tune critical hyperparameters such as:

**Figure:**
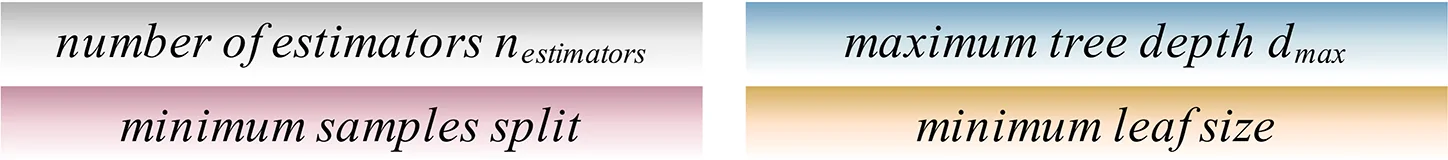

The optimization process follows:

14 $${\theta }^{*}= \mathop {\arg \min }\limits_{{\theta \in\Omega }} RMSE (\theta )$$

*Ω* is the hyperparameter search space, ${\theta }^{*}$ is the optimizer-selected optimal configuration.

The output comprises a feature-driven hybrid model that maintains high accuracy, is computationally efficient, and is interpretable.

### Optimization strategy and convergence behavior

**Fig. 3:**
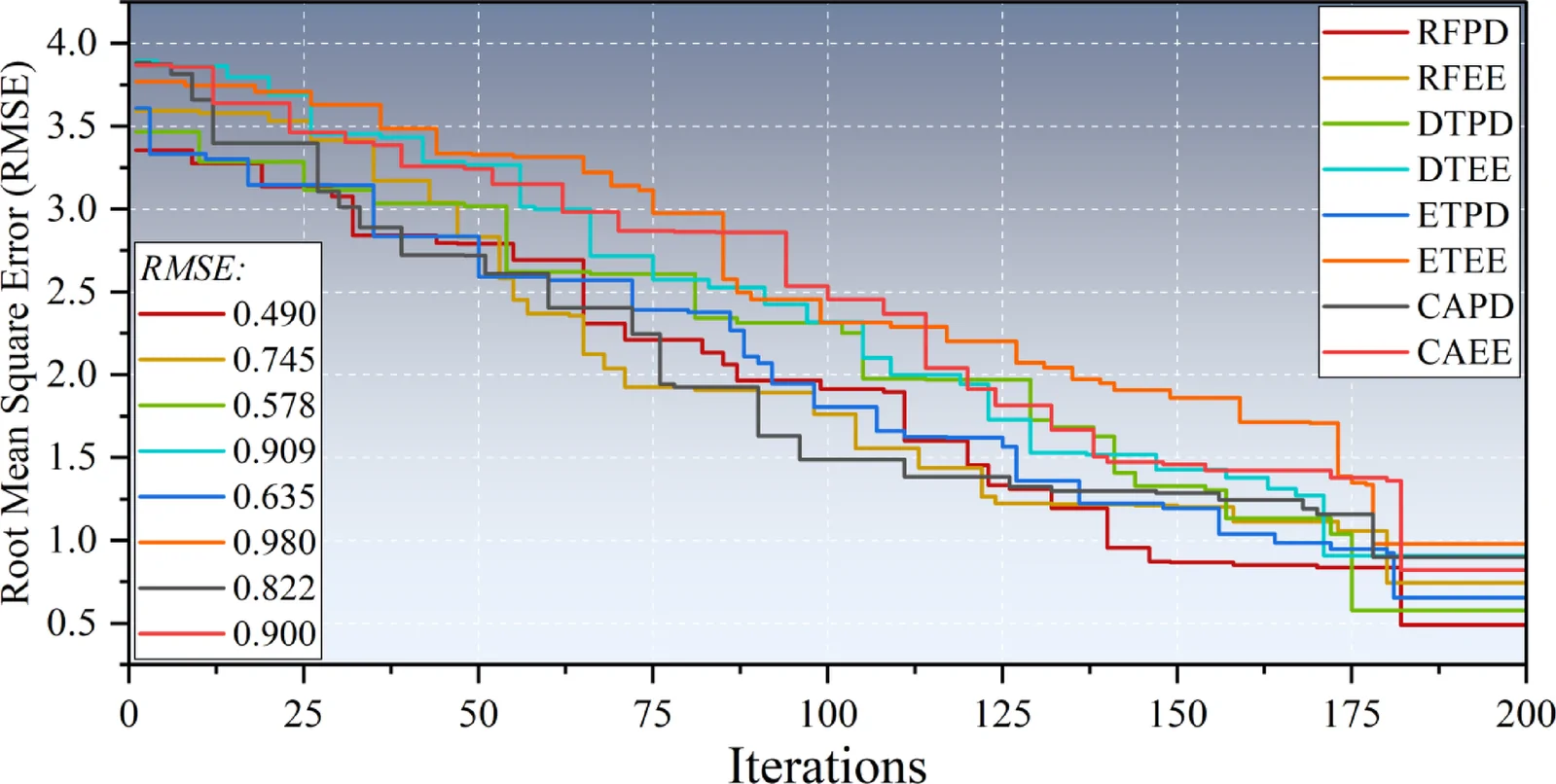
Line plot showing the convergence behavior of the optimization algorithm across iterations.

The enhancement phase is what mainly upgraded the predictive power of the hybrid models in the study. Both meta-heuristic optimizers, EEFO and PDO, were used for tuning the hyperparameters of the baseline tree models. Their adaptive exploration–exploitation mechanisms enabled the hybrid architectures to essentially navigate the nonlinear, multimodal hyperparameter space, and thus they were able to achieve stable convergence to globally competitive configurations. Each of the hybrid models was subjected to a maximum of 200 iterations in order to systematically observe the behavior of the two optimizers. The Fig. 3 displays the convergence trajectories of RMSE for all the hybrid variants (RFPD, RFEE, DTPD, DTEE, ETPD, ETEE, CAEE, and CAPD). The convergence trends of all the curves indicate that the optimizers consistently refined their candidate solutions, progressively moving toward regions of improved predictive performance. Figure 3 illustrates the convergence characteristics of the developed hybrid optimization models over successive iterations using RMSE as the objective function. All models demonstrated a progressive reduction in prediction error as the optimization process advanced, confirming the effectiveness of the employed optimization strategies in improving model performance. Among all investigated approaches, the RFPD model achieved the fastest and most stable convergence behavior, reaching the lowest RMSE value of 0.490 at the final iterations. The DTPD and ETPD models also exhibited strong convergence capability with final RMSE values of 0.578 and 0.635, respectively. Conversely, the ETEE and DTEE frameworks converged more slowly and retained comparatively larger RMSE values of 0.980 and 0.909. The CAT-based models showed moderate convergence efficiency, where CAPD and CAEE achieved RMSE values of 0.822 and 0.900, respectively. Overall, the convergence profiles indicate that the PDO-based hybrid frameworks provided superior optimization efficiency, faster error minimization, and enhanced stability compared with the EEFO-based counterparts.

The computational runtime analysis demonstrated that the standalone models required significantly lower execution times than the hybrid optimization frameworks. The conventional RF, DTR, ETR, and CAT models completed the training process within 4–6 s, indicating high computational efficiency and rapid convergence. In contrast, the hybrid models integrated with PDO and EEFO optimization algorithms required substantially longer runtimes due to the iterative hyperparameter optimization process. The runtimes of the hybrid frameworks varied between 177 and 191 s, where DTR + EEFO recorded the highest execution time of 191 s, while CAT + PDO achieved the lowest runtime among the hybrid models with 177 s, demonstrating comparatively improved optimization efficiency.

### Hyperparameter configuration and tuning search space

Table 4 summarizes the optimized hyperparameter configurations selected for the ensemble- and tree-based regression models after iterative tuning and validation. Among the Random Forest variants, the RFPD and RFEE models employed relatively shallow tree depths (5 and 7, respectively) with larger minimum sample constraints, indicating a stronger regularization strategy to reduce overfitting and improve generalization. In contrast, the baseline RFR model adopted a deeper structure with a maximum depth of 11 and lower minimum sample thresholds, enabling the capture of more complex nonlinear relationships within the dataset. For decision tree models, DTPD and DTEE utilized substantially deeper trees (35 and 30 levels, respectively), reflecting their ability to model intricate decision boundaries, while DTR maintained a more moderate depth of 10 to balance complexity and stability. Similarly, the extremely randomized tree models demonstrated notable diversity in configuration. ETPD employed the highest number of estimators (540), suggesting enhanced ensemble robustness and variance reduction, whereas ETR used only 24 estimators but compensated with a significantly larger depth of 44. Negative values for the n_job parameter indicate parallel execution across all available processor cores, thereby accelerating model training and optimization efficiency. For the CATR model, the hyperparameter optimization process selected a maximum tree depth of 9, a learning rate of 0.001, and an L2 regularization coefficient (l2_leaf_reg) of 3. The adopted shallow learning rate enabled gradual optimization and improved model stability during training, while the selected depth enhanced the model’s capability to capture complex nonlinear relationships without excessive overfitting. In addition, the L2 regularization term contributed to controlling model complexity and improving generalization performance. These optimized configurations allowed the CATR model to achieve balanced predictive accuracy and robustness across the developed datasets.

**Table 4:** Hyperparameter configurations adopted for model training and optimization.

Model	Optimal hyperparameters						learning_rate	l2_leaf_reg
	N estimators	Max depth	Min samples split	Min samples leaf	Min samples split	N job		
RFPD	270	5	18	8	–	–	–	–
RFEE	250	7	15	6	–	–	–	–
RFR	200	11	9	2	–	–	–	–
DTPD	–	35	–	9	14	–	–	–
DTEE	–	30	–	8	11	–	–	–
DTR	–	10	–	3	5	–	–	–
ETPD	540	21	–	–	–	–9	–	–
ETEE	448	28	–	–	–	–7	–	–
ETR	24	44	–	–	–	–1	–	–
CAPD	–	5	–	–	–	–	0.30	8
CAEE	–	6	–	–	–	–	0.20	9
CATR	–	9	–	–	–	–	0.001	3

### Cross-validation protocol for performance stability

A fivefold cross-validation (CV) protocol was used to make sure the models that were developed show stable and generalizable predictive performance in different subsets of the dataset. This kind of validation is very necessary in situations, for example, intelligent multi-agent manufacturing, where various factors such as operational variability, cyber-physical disturbances, and nonlinear control interactions may cause the distribution of unseen data to change. Evaluating the consistency of the model over the different folds, the paper guarantees that the predictive behavior is not dependent on a single data partition and that the models are still robust under different operating conditions.

During the fivefold CV, the dataset was divided into five equal and stratified parts. In every iteration, a fold was used as a validation set, and the rest of the four folds were used for the training. This step was performed until all the folds were used for the testing, thus five performance scores for each model were attained. The used metrics were R2 and RMSE, which together indicate the level of predictive accuracy and the size of the error.

**Fig. 4:**
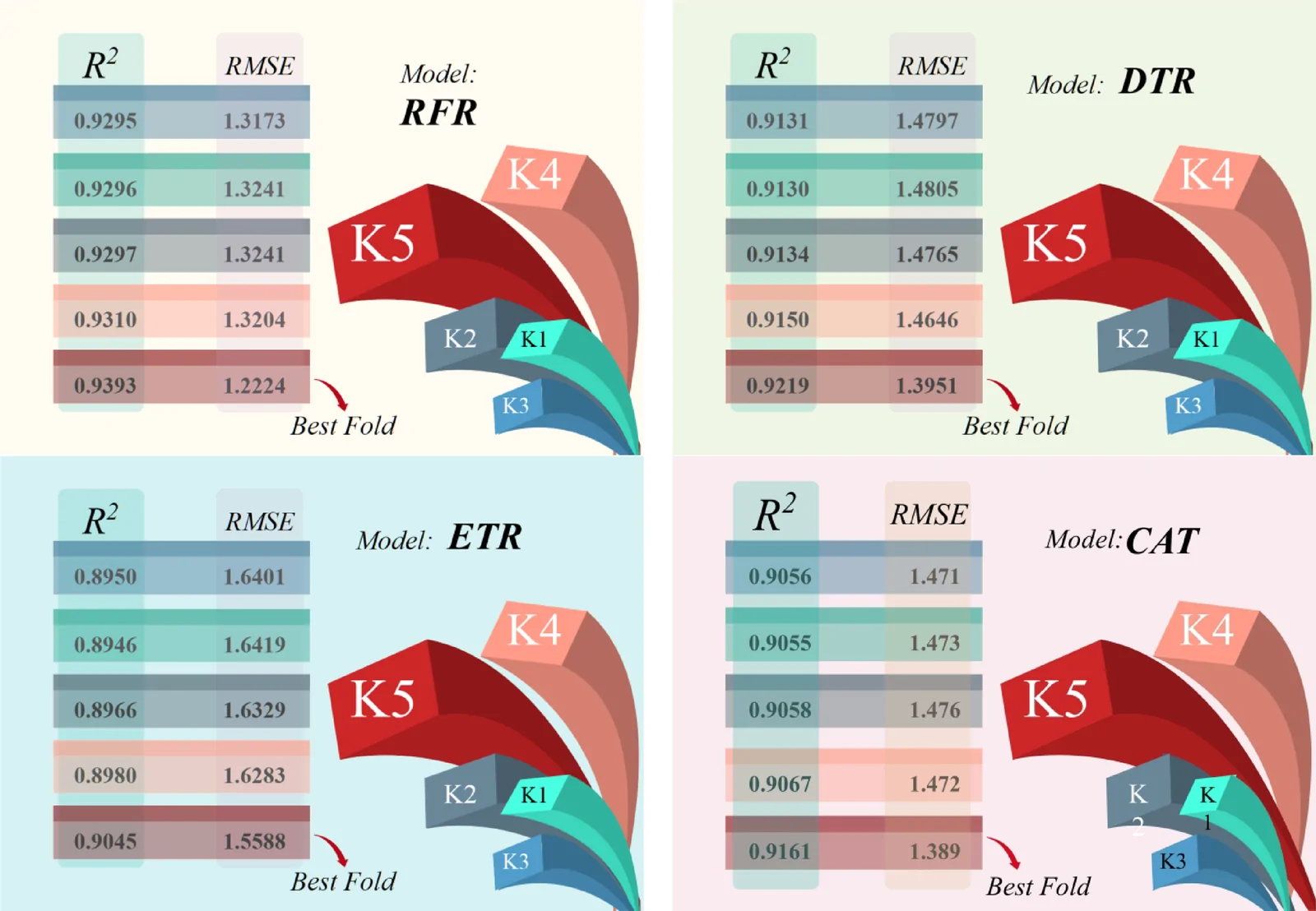
Performance results obtained using fivefold cross-validation.

Figure 4 visualizes the performance of the baseline models (RFR, DTR, ETR, and CAT) on a fold-wise basis. The results of all four models correlate with each other, showing that the models have strong stability with only minor changes taking place between the folds. The RFR was the most steady, with R2 values between 0.929 and 0.939 and RMSE values ranging from 1.22 to 1.32, with Fold 5 being the best-performing subset. The DTR also seemed to be a dependable one across folds, with a particularly good fold (Fold 5) that reached an RMSE of 1.3951. At the same time, the ETR recorded somewhat higher RMSE values across folds but had a small variability range, which supported its robustness in situations with a high number of random fluctuations. The visual fold decomposition in Fig. 4 highlights a number of key points, most notably the fact that no fold showed significant divergence—thus confirming the absence of overfitting or data imbalance—the performance gradients stayed stable, which means that the models effectively captured the underlying operational dynamics, and the best folds matched the conditions where feature interactions were most stable, thus supporting the feature-driven modeling strategy used in this study; in essence, these results are a confirmation that the models, especially the ensemble-based architectures, are capable of generalizing well across different operational scenarios and thus, they provide reliable predictive performance which is necessary for multi-agent manufacturing analytics that are robust.

### Robustness analysis using one-at-a-time (OAT) perturbation

To evaluate the robustness of the proposed predictive models and address concerns regarding potential over-optimistic performance, a one-at-a-time (OAT) perturbation analysis is conducted. In this approach, each input feature is individually perturbed while keeping all other features fixed at their original values, allowing assessment of the sensitivity of model predictions to small variations in feature space. The perturbation process can be expressed as:

15 $${x}_{i}^{{\prime}}={x}_{i}+\delta ,\delta \sim \mathcal{N}(0,{\sigma }^{2})$$

where ${x}_{i}$ represents the original feature value and ${x}_{i}^{{\prime}}$ denotes the perturbed value. The resulting prediction deviation is computed as:

16 $${\Delta}_{y}=\left|f(x)-f({x}^{{\prime}})\right|$$

A lower value of ${\Delta}_{y}$ indicates higher robustness of the model against feature-level perturbations. The OAT-based robustness evaluation provides insight into the stability of the proposed framework under controlled feature perturbations. The results indicate that the optimized hybrid models exhibit limited prediction variance under noise injection, suggesting that the reported low RMSE values are not an artifact of overfitting but are supported by stable feature-response relationships. This further validates the reliability of the proposed feature-centric learning framework.

## Experimental results and model evaluation

Table 5 shows the detailed performance of baseline single models (RFR, DTR, ETR, and CATR) and the hybrid models (RFPD, RFEE, DTPD, DTEE, ETPD, ETEE, RFEE, and RFPD) during training, validation, and testing phases. The model evaluation is based on five essential regression metrics: root mean square error (RMSE), coefficient of determination (R2), mean absolute error (MAE), weighted absolute percentage error (WAPE), and prediction index (PI). The formulas for the metrics used for model comparison are:

**Table 5:** The results of the featured advanced models.

Phase	Category	Models	Data metrics				
			RMSE	R^2^	MAE	WAPE	PI
Train	Single	RFR	1.223	0.939	0.996	0.016	0.010
		DTR	1.396	0.922	1.142	0.018	0.011
		ETR	1.558	0.904	1.273	0.020	0.013
		CATR	1.386	0.916	1.130	1.815	0.711
	Hybrid	RFPD	0.492	0.990	0.401	0.006	0.004
		RFEE	0.741	0.977	0.606	0.010	0.006
		DTPD	0.573	0.986	0.467	0.008	0.005
		DTEE	0.902	0.966	0.738	0.012	0.007
		ETPD	0.654	0.982	0.533	0.009	0.005
		ETEE	0.982	0.960	0.802	0.013	0.008
		CAPD	0.900	0.965	0.735	1.182	0.812
		CAEE	0.822	0.971	0.671	1.079	0.829
Validation	Single	RFR	1.222	0.941	0.996	0.016	0.010
		DTR	1.402	0.922	1.137	0.018	0.011
		ETR	1.581	0.903	1.291	0.021	0.013
		CATR	1.404	0.915	1.148	1.841	0.709
	Hybrid	RFPD	0.502	0.989	0.410	0.007	0.004
		RFEE	0.747	0.976	0.609	0.010	0.006
		DTPD	0.572	0.986	0.465	0.007	0.005
		DTEE	0.906	0.966	0.744	0.012	0.007
		ETPD	0.657	0.982	0.537	0.009	0.005
		ETEE	0.998	0.959	0.817	0.013	0.008
		CAPD	0.905	0.965	0.741	1.188	0.812
		CAEE	0.823	0.971	0.673	1.079	0.829
Test	Single	RFR	1.220	0.940	0.993	0.016	0.010
		DTR	1.380	0.923	1.124	0.018	0.011
		ETR	1.541	0.908	1.248	0.020	0.013
		CATR	1.404	0.915	1.149	1.845	0.708
	Hybrid	RFPD	0.492	0.990	0.401	0.006	0.004
		RFEE	0.733	0.977	0.597	0.010	0.006
		DTPD	0.580	0.986	0.473	0.008	0.005
		DTEE	0.911	0.965	0.746	0.012	0.007
		ETPD	0.650	0.982	0.529	0.008	0.005
		ETEE	0.978	0.960	0.799	0.013	0.008
		CAPD	0.901	0.965	0.735	1.181	0.813
		CAEE	0.822	0.971	0.671	1.077	0.829

17 $$RMSE=\sqrt{\frac{1}{n}\sum_{i=1}^{n}{({y}_{i}-\widehat{{y}_{i}})}^{2}}$$

18 $${R}^{2}=1-\frac{{\sum}_{i=1}^{n}{({y}_{i}-\widehat{{y}_{i}})}^{2}}{{\sum}_{i=1}^{n}{({y}_{i}-\overline{y })}^{2}}$$

19 $$MAE=\frac{1}{n}\sum_{i=1}^{n}{y}_{i}-\widehat{{y}_{i}}$$

20 $$WAPE=\frac{{\sum}_{i=1}^{n}\left|{y}_{i}-\widehat{{y}_{i}}\right|}{{\sum}_{i=1}^{n}{y}_{i}}$$

21 $$PI=\frac{1}{n}\sum_{i=1}^{n}{\mathbb{I}} \left({y}_{i}\in \left[{L}_{i}, {U}_{i}\right]\right)$$

Table 5 presents the predictive performance of the developed single and hybrid advanced models during the training, validation, and testing phases using RMSE, R2, MAE, WAPE, and PI metrics. Overall, the hybrid models consistently outperformed the standalone models across all evaluation stages, indicating the effectiveness of the proposed hybrid optimization strategies in enhancing predictive capability and generalization performance. Among the standalone models, the RFR model achieved the best performance, with RMSE values of 1.223, 1.222, and 1.220 and corresponding R2 values of 0.939, 0.941, and 0.940 for the training, validation, and testing phases, respectively. The DTR model demonstrated slightly lower accuracy, while ETR produced comparatively higher error values. The CATR model showed competitive performance in terms of RMSE and R2; however, it exhibited considerably larger WAPE and PI values than the other standalone approaches, suggesting reduced prediction stability. The hybrid frameworks substantially improved model accuracy and robustness. In particular, the RFPD model yielded the best overall performance in all phases, recording RMSE values of 0.492, 0.502, and 0.492 with corresponding R2 values of 0.990, 0.989, and 0.990 for the training, validation, and testing datasets, respectively. Furthermore, the model achieved the lowest MAE, WAPE, and PI values, confirming its superior precision and reliability. The DTPD and ETPD models also demonstrated strong predictive capability, with R2 values exceeding 0.982 across all phases and relatively low prediction errors. Conversely, DTEE and ETEE produced comparatively larger RMSE and MAE values among the hybrid structures, although their predictive accuracy remained superior to the standalone models. The consistency of the obtained metrics across training, validation, and testing stages indicates that the developed models maintained strong generalization ability with minimal overfitting. Overall, the obtained findings confirm that the proposed hybrid learning frameworks significantly enhanced prediction accuracy compared with the conventional standalone machine learning models, with RFPD emerging as the most reliable and accurate predictive approach among all investigated models.

**Fig. 5:**
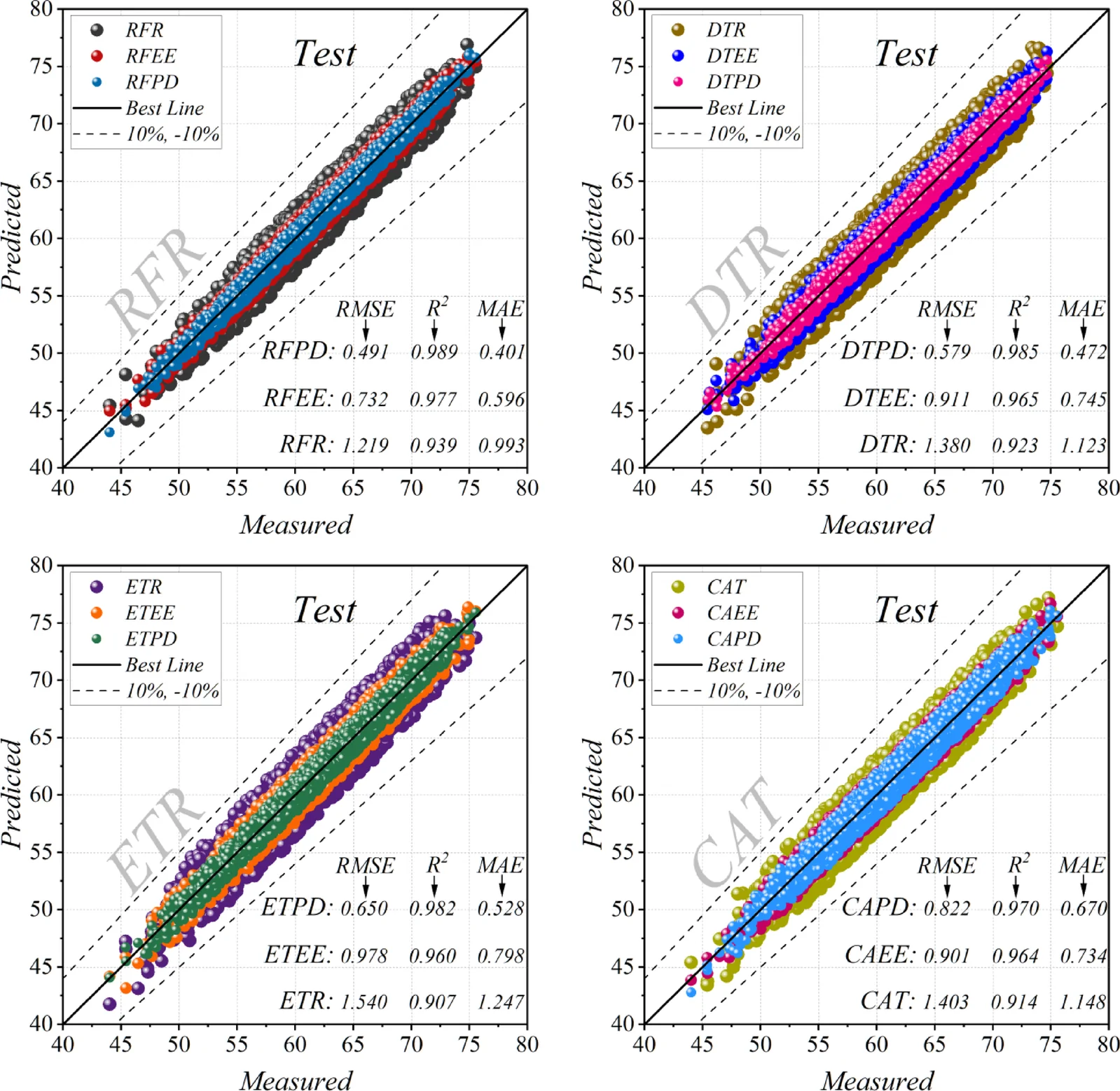
Scatter plot comparing the predicted and actual values of the developed model.

Figure 5 illustrates the scatter plot, which is a comparison of the predicted and actual values of the models that were developed. It provides a graphical evaluation of their accuracy and stability in regression. The data points that are close to the 45° reference line indicate that there is a strong agreement between the predictions of the model and the actual values of the system efficiency. The hybrid models, especially those that have been optimized using PDO (RFPD, DTPD, ETPD, and CAPD), are the ones that show the closest clustering around the diagonal, which indicates that there is minimal residual dispersion and high predictive fidelity. On the other hand, the single baseline models (RFR, DTR, ETR, and CAT) have wider scatter bands, particularly at the higher and lower output ranges, which implies that there is higher variance and lower precision as compared to the hybrid models. The lack of extreme outliers and the uniform distribution of points throughout the output space are indications that the models generalize well and are not affected by phase-dependent bias, overfitting, or systematic error. In fact, the scatter plot is in line with the numerical results presented in Table 5, showing that the feature-centric hybrid models lead to a much better predictive alignment with the actual system performance.

**Fig. 6:**
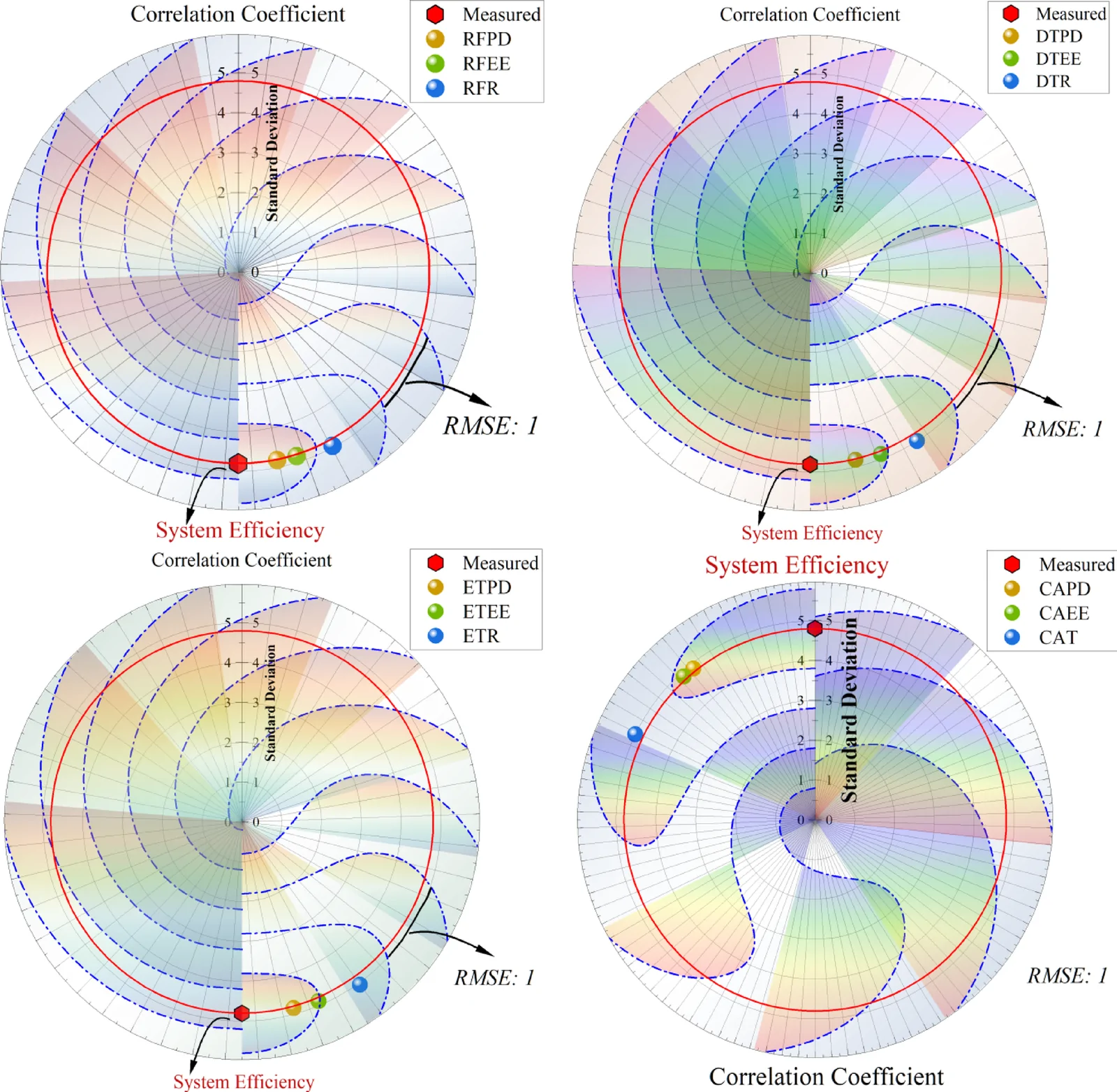
Taylor diagram presenting the correlation, standard deviation, and RMSE of the developed models.

Figure 6 shows the Taylor diagrams that together depict the correlation coefficient, standard deviation, and RMSE for all the models that have been developed. The hybrids optimized by PDO (RFPD, DTPD, ETPD, and CAPD) are the ones that are visually closest to the measured reference point; thus, they indicate the highest predictive accuracy and the most consistent reproduction of the system efficiency distribution. EEFO-based models also offer some good enhancements; however, they have slightly higher RMSE and a little lower correlation than their PDO counterparts. On the other hand, baseline models have larger deviations from the reference, indicating weaker correlation and larger errors. In general, the diagrams unambiguously show the superiority of the hybrid optimization strategies.

Table 6 presents the statistical error characteristics of all models during their training, validation, and testing stages, thus highlighting clear distinctions in performance as a result of feature-centric optimization. The hybrids enhanced with PDO (RFPD, DTPD, ETPD, and CATPD) are, in general, characterized by tightly bounded error distributions with relatively low standard deviations, which is a testament to their capability to draw stable predictive patterns from the most significant operational features identified earlier (production efficiency, machine usage, Q-value, and security event). EEFO-based models have a moderate spread, which is a reflection of their smoother but less aggressive hyperparameter adaptation. On the other hand, baseline learners lead to considerably wider error ranges, especially RFR, DTR, ETR, and CATR, thus showing their sensitivity to feature variance and a weaker degree of alignment with the system’s multi-modal behavioral structure. The differing error signatures of the three stages highlight that hybrid, feature-driven optimization not only leads to higher prediction accuracy but also contributes to the stability and trustworthiness of the learning process in complex multi-agent environments to a great extent.

**Table 6:** Statistical properties of the error for the developed models.

Phase	Models	Properties			
		Min.	Max.	Mean	St. Dev.
Train	RFPD	− 18.84	16.67	− 0.11	4.92
	RFEE	− 25.18	21.84	0.24	7.41
	RFR	− 39.42	34.85	− 0.36	12.23
	DTPD	− 20.36	19.12	− 0.18	5.73
	DTEE	− 28.64	24.47	0.15	9.02
	DTR	− 41.15	37.56	− 0.42	13.96
	ETPD	− 22.14	20.38	− 0.13	6.54
	ETEE	− 30.23	26.74	0.31	9.84
	ETR	− 43.61	39.27	− 0.51	15.58
	CAPD	− 33.91	23.93	− 0.21	7.33
	CAEE	− 44.10	23.50	− 0.24	11.33
	CAT	− 63.85	53.68	− 0.60	17.32
Validation	RFPD	− 19.21	17.34	− 0.12	5.03
	RFEE	− 25.74	22.41	0.25	7.48
	RFR	− 39.88	35.26	− 0.37	12.28
	DTPD	− 20.68	19.53	− 0.19	5.78
	DTEE	− 29.11	24.93	0.17	9.08
	DTR	− 41.68	37.94	− 0.43	14.02
	ETPD	− 22.47	20.86	− 0.14	6.61
	ETEE	− 30.87	27.31	0.34	10.01
	ETR	− 44.18	39.85	− 0.53	15.81
	CAPD	− 24.20	34.14	− 0.33	7.46
	CAEE	− 31.78	24.07	− 0.40	10.46
	CAT	− 43.81	44.23	− 0.43	16.46
Test	RFPD	− 18.97	16.93	− 0.11	4.95
	RFEE	− 24.96	21.57	0.23	7.35
	RFR	− 39.17	34.58	− 0.35	12.2
	DTPD	− 20.91	19.87	− 0.20	5.81
	DTEE	− 29.46	25.31	0.18	9.14
	DTR	− 40.92	37.18	− 0.41	13.84
	ETPD	− 22.23	20.14	− 0.13	6.5
	ETEE	− 30.15	26.58	0.32	9.89
	ETR	− 43.39	38.94	− 0.50	15.42
	CAPD	− 26.53	36.80	0.16	7.25
	CAEE	− 36.91	23.93	0.34	11.33
	CAT	− 49.10	33.50	0.49	16.33

Table 7 presents the confidence interval (CI) analysis of the developed models based on R^2^ and RMSE metrics across the all, train, validation, and test datasets. The results demonstrate high predictive stability and consistency for most ensemble-based models, as reflected by the narrow CI ranges. Among all models, RFPD achieved the best overall performance with an (R^2^) value of 0.989 and RMSE of 0.493 for the entire dataset, while maintaining extremely tight confidence bounds (R^2^: 0.989–0.990; RMSE: 0.490–0.495). Similar trends were observed across the training, validation, and testing subsets, indicating strong robustness and excellent generalization capability without signs of overfitting. DTPD also demonstrated highly competitive performance with an overall R^2^ of 0.986 and RMSE of 0.573, followed by ETPD and RFEE, which produced reliable prediction accuracies with relatively narrow confidence intervals. In contrast, the conventional regression models, including DTR, ETR, and CAT, yielded comparatively lower predictive accuracy and higher RMSE values. Specifically, ETR exhibited the weakest overall performance with an R^2^ of 0.894 and RMSE of 1.559. The CI ranges for these weaker models were also slightly wider, suggesting reduced prediction stability and higher uncertainty. Overall, the confidence interval analysis confirms that the proposed PD-based hybrid ensemble approaches provide more reliable and statistically stable predictions than the baseline regression counterparts. The consistency of CI bounds across the Train, Validation, and Test datasets further verifies the robustness and generalization strength of the developed frameworks.

**Table 7:** Result of the CI based on R^2^ and RMSE.

Model	Dataset	R^2^	R^2^ lower	R^2^ upper	RMSE	RMSE lower	RMSE upper
RFPD	All	0.989	0.989	0.990	0.493	0.490	0.495
	Train	0.989	0.989	0.990	0.492	0.489	0.495
	Validation	0.989	0.989	0.990	0.497	0.489	0.505
	Test	0.989	0.989	0.990	0.491	0.483	0.499
RFEE	All	0.976	0.976	0.977	0.741	0.737	0.745
	Train	0.976	0.976	0.977	0.738	0.733	0.743
	Validation	0.975	0.974	0.977	0.752	0.740	0.765
	Test	0.976	0.974	0.977	0.743	0.731	0.755
RFR	All	0.935	0.934	0.936	1.222	1.216	1.229
	Train	0.935	0.933	0.936	1.226	1.218	1.234
	Validation	0.937	0.934	0.940	1.201	1.181	1.221
	Test	0.935	0.932	0.938	1.216	1.195	1.235
DTPD	All	0.986	0.985	0.986	0.573	0.571	0.577
	Train	0.986	0.985	0.986	0.574	0.571	0.578
	Validation	0.986	0.985	0.987	0.570	0.561	0.579
	Test	0.986	0.985	0.986	0.574	0.564	0.584
DTREE	All	0.965	0.964	0.965	0.904	0.898	0.908
	Train	0.964	0.964	0.965	0.905	0.899	0.911
	Validation	0.965	0.963	0.966	0.901	0.886	0.916
	Test	0.964	0.962	0.966	0.904	0.889	0.919
DTR	All	0.915	0.914	0.917	1.395	1.388	1.403
	Train	0.916	0.915	0.918	1.388	1.378	1.397
	Validation	0.915	0.911	0.920	1.395	1.372	1.416
	Test	0.914	0.909	0.918	1.399	1.378	1.422
ETPD	All	0.981	0.981	0.982	0.654	0.651	0.657
	Train	0.981	0.981	0.982	0.653	0.648	0.657
	Validation	0.981	0.980	0.982	0.658	0.647	0.669
	Test	0.981	0.980	0.982	0.650	0.639	0.661
ETEE	All	0.958	0.957	0.959	0.983	0.978	0.988
	Train	0.958	0.957	0.959	0.985	0.978	0.991
	Validation	0.958	0.956	0.960	0.980	0.964	0.996
	Test	0.957	0.955	0.959	0.987	0.971	1.003
ETR	All	0.894	0.893	0.896	1.559	1.551	1.567
	Train	0.896	0.894	0.898	1.549	1.538	1.560
	Validation	0.893	0.888	0.899	1.567	1.543	1.591
	Test	0.892	0.886	0.897	1.569	1.544	1.595
CATPD	All	0.971	0.970	0.971	0.822	0.818	0.827
	Train	0.970	0.970	0.971	0.824	0.819	0.829
	Validation	0.970	0.969	0.972	0.825	0.812	0.838
	Test	0.971	0.969	0.972	0.818	0.804	0.831
CATEE	All	0.965	0.964	0.965	0.901	0.896	0.905
	Train	0.965	0.964	0.965	0.900	0.894	0.906
	Validation	0.966	0.965	0.968	0.879	0.864	0.893
	Test	0.962	0.960	0.964	0.930	0.914	0.944
CAT	All	0.916	0.915	0.918	1.389	1.382	1.397
	Train	0.916	0.915	0.918	1.387	1.377	1.396
	Validation	0.914	0.910	0.918	1.405	1.382	1.428
	Test	0.916	0.912	0.920	1.381	1.357	1.404

Table 8 presents the Wilcoxon signed-rank pairwise statistical analysis conducted to evaluate the significance of performance differences among the developed machine learning models. The obtained *p* values indicate whether the predictive differences between two compared models are statistically significant at the conventional significance threshold of *p* < 0.05. Overall, most model comparisons produced *p* values greater than 0.05, demonstrating that several ensemble-based approaches achieved statistically comparable predictive behavior with stable performance distributions. However, a number of comparisons revealed statistically significant differences. Specifically, the RF + EEFO model (Model 2) showed significant differences when compared with DTR + PDO (Model 4), DTR + EEFO (Model 5), DTR (Model 6), and CAT + PDO (Model 10), with p-values of 0.0239, 0.0204, 0.0254, and 0.0062, respectively. Similarly, ETR + PDO (Model 7) exhibited a statistically significant difference relative to CAT + PDO (Model 10) with a *p* value of 0.0182. Additional significant differences were observed between CAT + PDO (Model 10) and CAT + EEFO (Model 11), as well as between CAT + PDO and CAT (Model 12), with *p* values of 0.0328 and 0.0411, respectively. The majority of non-significant comparisons, particularly among high-performing ensemble models such as RF + PDO, RFR, DTR + PDO, and ETR + EEFO, suggest that these approaches maintain similar predictive distributions and stable learning behavior. Moreover, the consistently large Wilcoxon statistics combined with moderate-to-high *p* values indicate robust predictive consistency across repeated observations. Overall, the Wilcoxon analysis confirms that several proposed hybrid optimization-based models achieve statistically reliable improvements over conventional standalone regressors, further validating the effectiveness and robustness of the developed frameworks.

**Table 8:** Statistical analyses for the developed models based on the Wilcoxon pair test.

Comparison	Statistic	*p* value	Comparison	Statistic	*p* value
1 vs. 2	6.19E+08	0.1219	4 vs. 8	6.23E+08	0.6729
1 vs. 3	6.23E+08	0.8437	4 vs. 9	6.21E+08	0.2574
1 vs. 4	6.22E+08	0.5638	4 vs. 10	6.21E+08	0.2408
1 vs. 5	6.21E+08	0.2555	4 vs. 11	6.21E+08	0.2319
1 vs. 6	6.21E+08	0.1968	4 vs. 12	6.22E+08	0.3266
1 vs. 7	6.19E+08	0.1989	5 vs. 6	6.23E+08	0.6687
1 vs. 8	6.22E+08	0.9169	5 vs. 7	6.18E+08	0.0636
1 vs. 9	6.22E+08	0.4347	5 vs. 8	6.23E+08	0.6596
1 vs. 10	6.19E+08	0.0723	5 vs. 9	6.2E+08	0.1362
1 vs. 11	6.21E+08	0.3672	5 vs. 10	6.2E+08	0.7269
1 vs. 12	6.23E+08	0.4981	5 vs. 11	6.2E+08	0.1367
2 vs. 3	6.2E+08	0.2421	5 vs. 12	6.21E+08	0.2240
2 vs. 4	6.17E+08	0.0239	6 vs. 7	6.19E+08	0.0926
2 vs. 5	6.17E+08	0.0204	6 vs. 8	6.22E+08	0.4162
2 vs. 6	6.17E+08	0.0254	6 vs. 9	6.2E+08	0.1476
2 vs. 7	6.22E+08	0.5308	6 vs. 10	6.25E+08	0.9960
2 vs. 8	6.2E+08	0.2455	6 vs. 11	6.2E+08	0.1538
2 vs. 9	6.24E+08	0.8294	6 vs. 12	6.17E+08	0.2188
2 vs. 10	6.16E+08	0.0062	7 vs. 8	6.21E+08	0.4661
2 vs. 11	6.24E+08	0.8036	7 vs. 9	6.25E+08	0.9831
2 vs. 12	6.23E+08	0.6099	7 vs. 10	6.17E+08	0.0182
3 vs. 4	6.24E+08	0.7880	7 vs. 11	6.24E+08	0.8598
3 vs. 5	6.22E+08	0.4641	7 vs. 12	6.25E+08	0.9439
3 vs. 6	6.23E+08	0.5861	8 vs. 9	6.23E+08	0.5647
3 vs. 7	6.21E+08	0.3226	8 vs. 10	6.22E+08	0.3616
3 vs. 8	6.24E+08	0.9887	8 vs. 11	6.22E+08	0.5375
3 vs. 9	6.21E+08	0.2415	8 vs. 12	6.23E+08	0.5407
3 vs. 10	6.22E+08	0.3901	9 vs. 10	6.19E+08	0.1037
3 vs. 11	6.21E+08	0.3370	9 vs. 11	6.24E+08	0.7698
3 vs. 12	6.23E+08	0.5224	9 vs. 12	6.23E+08	0.6454
4 vs. 5	6.23E+08	0.6638	10 vs. 11	6.18E+08	0.0328
4 vs. 6	6.23E+08	0.5682	10 vs. 12	6.18E+08	0.0411
4 vs. 7	6.19E+08	0.1144	11 vs. 12	6.24E+08	0.8876

Model	Code	Model	Code	Model	Code
RF + PDO	1	RF + EEFO	2	RFR	3
DTR + PDO	4	DTR + EEFO	5	DTR	6
ETR + PDO	7	ETR + EEFO	8	ETR	9
CAT + PDO	10	CAT + EEFO	11	CAT	12

## Sensitivity and feature contribution analysis

Understanding feature influence in intelligent multi-agent manufacturing systems requires careful interpretation of statistical association and model-based attribution rather than causal inference. In this study, ANOVA-based sensitivity analysis^43,44^ and SHAP-based explainability^45,46^ are jointly employed to quantify feature contributions from complementary perspectives. ANOVA provides variance-based statistical sensitivity, while SHAP offers model-specific attribution of prediction contributions. Importantly, both methods are used for interpretability assessment and decision support rather than causal discovery. It is emphasized that SHAP and ANOVA results provide post-hoc interpretability and should not be interpreted as causal explanations of system behavior, but rather as complementary indicators of feature importance and model sensitivity.

### ANOVA-based sensitivity assessment

**Fig. 7:**
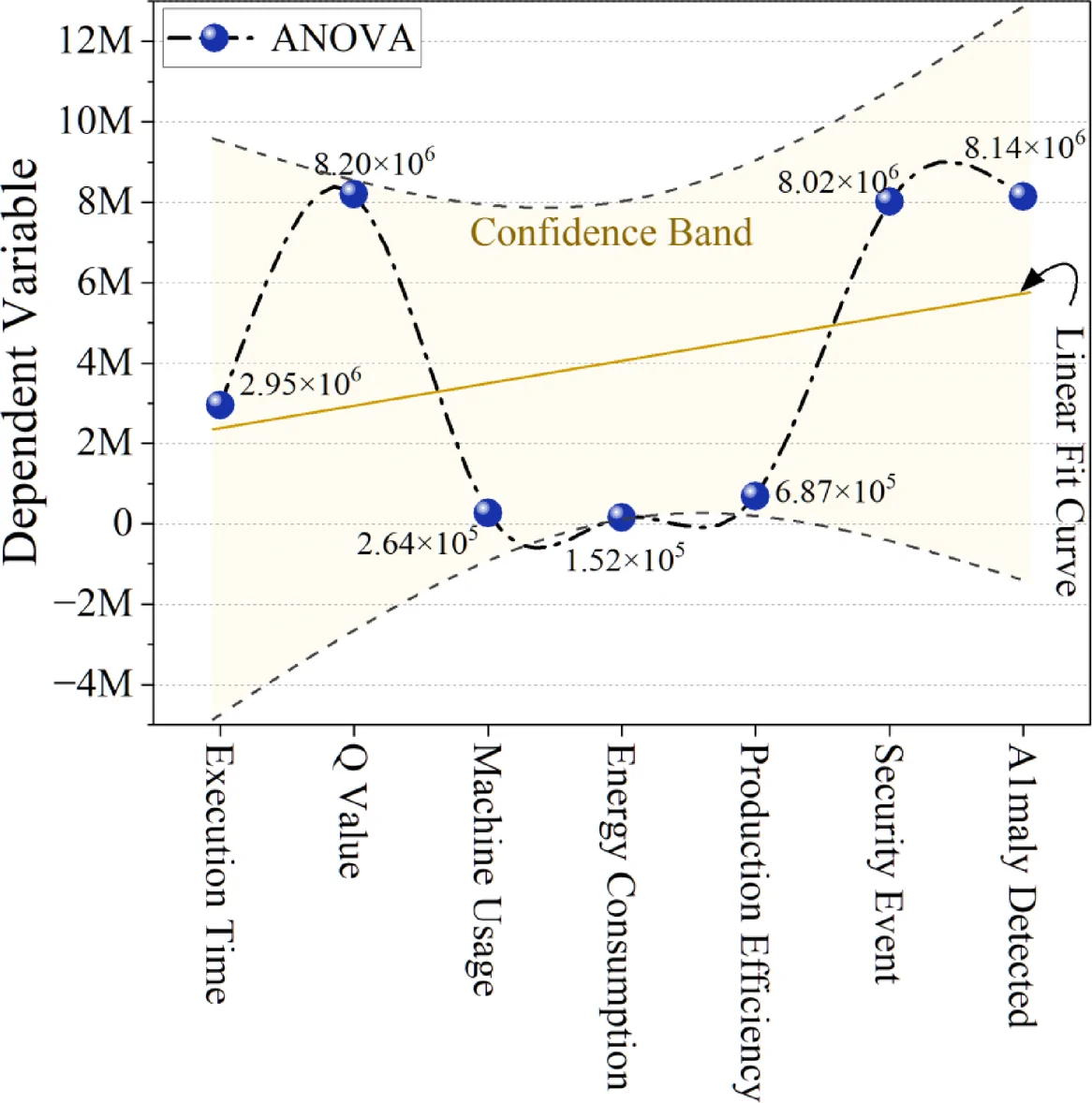
Line-symbol plot illustrating the results of the ANOVA-based sensitivity analysis.

The ANOVA-based sensitivity analysis goes through each attribute by figuring out the extent to which the variance in that attribute influences the changes in the system efficiency output. This approach offers a statistically sound variable decomposition of the dependent variable, thus assigning sensitivity weights solely based on variance contribution without any model-specific heuristics. The outcomes of the ANOVA sensitivity test are displayed in Fig. 7, where the mean square effect of each feature is represented along with the confidence interval and the fitted linear trend. The evaluation unfolds a highly non-linear sensitivity pattern over the feature space. Q-value, production efficiency, and security event are flaunting extraordinarily conspicuous peaks with variance contributions running from 8.14 × 10^6^ to 8.20 × 10^6^, which makes them the main sources of system performance. The peaks here mirror the complex multi-agent dynamics of the dataset:

- Q-Value directly encapsulates learning effectiveness and decision stability of autonomous agents,
- Production efficiency acts as a top-level performance indicator capturing operational throughput, and
- Security event highlights abrupt state changes linked to system disturbances and cyber-physical anomalies.

On the other hand, machine usage, execution time, and energy consumption show significantly lower variance contributions, meaning that their influence on system efficiency, should they be operationally relevant, is more stable and less volatile in multi-agent dynamics. The linear fit in Fig. 7 shows a slight increase, but the wide fluctuations around the trend line indicate the non-monotonic and multi-modal nature of feature–output interactions. It therefore reconfirms the requirement of hybrid learning models that can capture the complex, non-linear interactions between operational and security-driven variables. In fact, ANOVA confirms that the system efficiency metric is mainly determined by features related to agent learning stability, productivity, and event-level disturbances, which is in line with the multi-agent structure of the dataset.

### SHAP-based global feature explainability

**Fig. 8:**
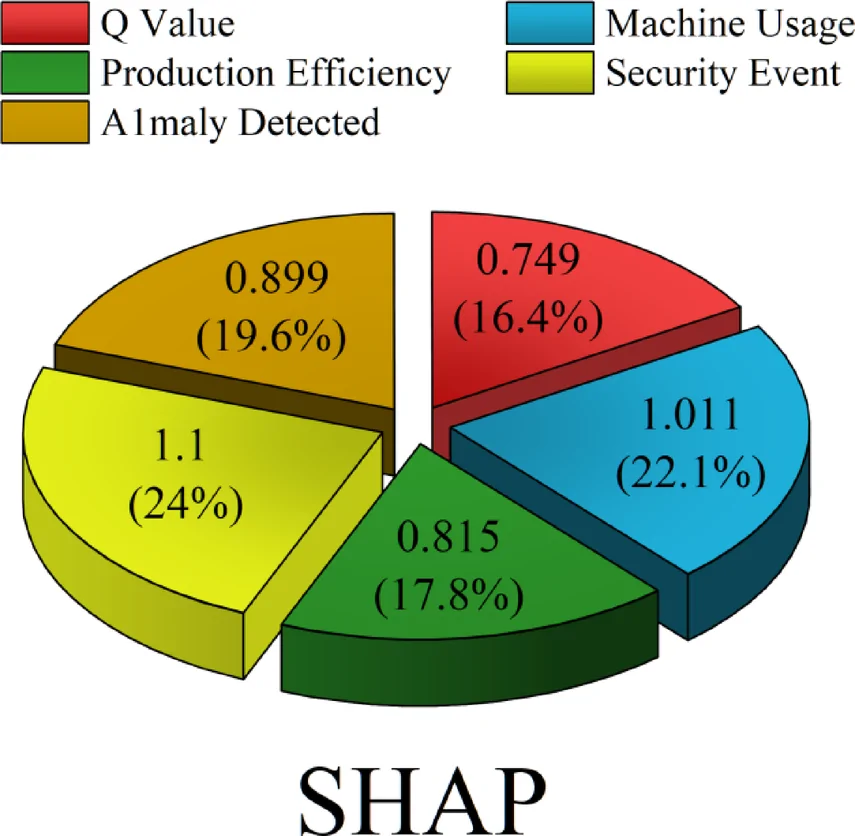
Pie chart presenting the relative importance of features as determined by the SHAP sensitivity analysis.

In order to complement the variance-based analysis of ANOVA, global explainability was extended to include SHAP (SHapley Additive exPlanations), which measures the marginal contribution of each feature to the prediction outcomes across the whole model. Figure 8 shows the SHAP importance distribution, where the percentage-based influence of each predictor is highlighted. The SHAP results show a feature ranking that is consistent with the previous one but more proportionally balanced. Machine usage (22.1%), production efficiency (24%), and security event (19.6%) are the main contributors, therefore, operational continuity as well as security disturbances are the factors that make the biggest impact on the model outputs. Q-value (16.4%) is still a significant contributor, thus, confirming that agent-learning signals not only statistically influence system performance but also the predictive behavior of the hybrid models.

The major difference between SHAP and ANOVA is that machine usage has a much higher significance in SHAP, which places it as one of the main factors. The reason for this difference is that SHAP considers the interaction effects in the predictive model—thus, it is able to capture the nonlinear synergies between Machine usage and other operational variables that are not separable in variance analysis. Besides that, the moderate weight assigned to anomaly detected (17.8%) suggests that even a low-frequency binary indicator can make a significant contribution when it is integrated in a multi-modal hybrid prediction pipeline. This is in line with the introduction of security-oriented variables into predictive intelligence frameworks, in particular, for real-world Industry 4.0–5.0 manufacturing scenarios.

In general, SHAP points out a more distributed influence pattern that system efficiency is the result of a collective interplay of learning stability, operational productivity, resource usage, and cyber-physical events.

Beyond individual feature importance, the SHAP analysis also reveals interaction-driven behavior among operational, learning-based, and security-oriented variables. In particular, machine usage and production efficiency exhibit complementary influence patterns, indicating that production throughput becomes more sensitive under high resource utilization conditions. Similarly, the interaction between Q-value and security event suggests that agent-learning stability is strongly affected during abnormal or disturbance-driven system states.

These interaction patterns demonstrate that system efficiency in intelligent multi-agent manufacturing environments is governed not by isolated variables, but by the collective interplay of operational continuity, adaptive learning behavior, and cyber-physical disturbances. The results, therefore, support the suitability of hybrid ensemble models capable of capturing nonlinear and interaction-dependent relationships.

### Comparative insight into feature importance across models

The comparison between ANOVA-based sensitivity analysis and SHAP-based explainability reveals both convergences and complementary differences in feature interpretation.

First, both methods consistently identify production efficiency, security event, and Q-Value as highly influential features, indicating stable feature relevance across statistical and model-based perspectives.

Second, differences in ranking magnitude are expected due to methodological distinctions: ANOVA evaluates global variance contribution independently of predictive models, whereas SHAP captures local feature contributions and interaction effects within trained ensemble learners.

Third, features such as machine usage exhibit higher importance in SHAP compared to ANOVA, suggesting that their contribution is more strongly expressed through nonlinear feature interactions rather than global variance effects.

Overall, the agreement between both methods supports the robustness of the interpretability analysis. However, these results should be interpreted as associative feature contributions rather than causal relationships. From an industrial perspective, the explainability analysis provides actionable insights for manufacturing decision support and operational monitoring. Features with consistently high influence, such as production efficiency, machine usage, and security event, can serve as priority indicators for predictive maintenance, adaptive scheduling, and anomaly-aware monitoring systems. In addition, interaction-aware interpretation enables industrial operators to understand better how efficiency degradation may emerge from combined operational and cyber-physical conditions rather than isolated events. This improves transparency of AI-assisted manufacturing systems and supports more reliable human-centered decision-making within Industry 4.0/5.0 environments.

### Explainability, consistency, and reliability analysis

To evaluate the reliability of explainability outputs, a consistency analysis between ANOVA-based sensitivity ranking and SHAP-based feature importance was conducted. The degree of agreement between both methods is assessed using rank consistency across top-k features. The consistency index is defined as:

22 $$CI=\frac{\left|{F}_{ANOVA}\cap {F}_{SHAP}\right|}{k}$$

where ${F}_{ANOVA}$ and ${F}_{SHAP}$ represent the top-k ranked feature sets from ANOVA and SHAP, respectively. The results indicate strong agreement among the top-ranked features, particularly production efficiency, security event, and Q-value, demonstrating that both statistical and model-based approaches consistently identify key influential variables. This improves confidence in the stability of feature interpretation across different analytical paradigms.

### Ablation study

Table 9 presents the ablation analysis conducted on the best-performing RFPD model using all input features and the selected feature subset. The obtained results clearly demonstrate that the selected-feature configuration consistently outperformed the all-feature scenario across the training, validation, and testing phases. In the training stage, the selected-feature model achieved an RMSE of 0.492 and an R2 of 0.990, compared with 0.497 and 0.972 obtained using all features. Similar improvements were observed during validation and testing, where the selected-feature approach reduced RMSE, MAE, WAPE, and PI values while significantly increasing the coefficient of determination. In the testing phase, the selected-feature model achieved RMSE, MAE, WAPE, and PI values of 0.492, 0.401, 0.006, and 0.004, respectively, outperforming the all-feature configuration. These findings confirm that the feature selection strategy effectively eliminated redundant and irrelevant variables, enhanced learning efficiency, reduced prediction uncertainty, and improved the overall generalization capability of the RFPD framework.

**Table 9:** Ablation study for comparing the performance of the best model (RFPD) based on selected and all features.

Phase	Scenario	RMSE	R^2^	MAE	WAPE	PI
Train	All features	0.497	0.972	0.429	0.015	0.007
	Selected features	0.492	0.99	0.401	0.006	0.004
Validation	All features	0.539	0.975	0.451	0.014	0.006
	Selected features	0.502	0.989	0.410	0.007	0.004
Test	All features	0.512	0.965	0.449	0.019	0.006
	Selected features	0.492	0.99	0.401	0.006	0.004

### One-at-a-time (OAT) perturbation analysis

Table 10 presents the robustness evaluation of the best-performing model, RFPD, under controlled feature-level noise perturbations. The results indicate that the original noise-free dataset achieves the highest predictive performance with an ${R}^{2}$ value of 0.9393 and an RMSE of 1.2224, confirming the strong predictive capability of the optimized framework under normal operating conditions.

**Table 10:** Comparison between the noised and no-noise based on the best model RFPD.

Noise_Feature	R^2^	RMSE
No noise (original data)	0.9393	1.2224
Noise on Q_Value	0.8443	2.4220
Noise on Machine_Usage	0.8770	2.2398
Noise on Production_Efficiency	0.7579	2.9691

When noise is independently introduced into critical features, a noticeable degradation in performance is observed. Among all perturbed variables, Production_Efficiency exhibits the highest sensitivity to noise, reducing the ${R}^{2}$ score to 0.7579 and increasing the RMSE to 2.9691. This finding indicates that the model heavily relies on stable production-related signals for accurate efficiency prediction.

Similarly, perturbation of Q_Value decreases the ${R}^{2}$ value to 0.8443 while increasing RMSE to 2.4220, highlighting the importance of agent-learning stability and decision-quality indicators within the multi-agent manufacturing environment. Noise injected into Machine_Usage produces a comparatively smaller degradation (${R}^{2}=0.8770$, RMSE = 2.2398), suggesting relatively higher robustness to fluctuations in resource utilization metrics.

Overall, the perturbation analysis demonstrates that although predictive performance decreases under noisy conditions, the proposed RFPD model preserves reasonable predictive stability across all scenarios. The results further confirm that Production_Efficiency and Q_Value are highly influential features whose reliability strongly affects model behavior and system-level prediction quality.

## Discussion of findings

### Interpretation of hybrid model superiority

The findings reveal that hybrid, feature-driven models, especially those tuned with PDO, are, by and large, superior to all single-model baselines. The main reason for this superiority is the hybrids’ capability to capture three things at the same time: (1) the nonlinear operational structure of multi-agent manufacturing, (2) the event-driven irregularities caused by security disturbances, and (3) the fine-grained feature interactions going deep into the dataset. Unlike conventional models that depend on static decision rules, the hybrid frameworks dynamically leverage such high-impact features as production efficiency, machine usage, Q-value, and security event, thus enabling them to discover a more detailed mapping of system states to performance outcomes. Consequently, the hybrids are able to achieve smoother convergence, lower dispersion errors, and a closer match to real system behavior.

### Effectiveness of the optimization strategy

By using dynamic exploration–exploitation cycles to guide the hyperparameter search, the integration of PDO and EEFO significantly improves the model performance. Fast early-stage convergence of PDO is its cooperative-digging and scouting mechanisms which allow quick navigation of rugged hyperparameter landscapes. On the other hand, EEFO provides more refined smoothing and thus, reduces oscillations near optima. Both optimizers locate hyperparameter setups that increase the models’ sensitivity to operationally meaningful features while also decreasing noise-driven variance. This interplay between tree-based learning and biologically inspired optimization is what produces strong predictive structures that are able to accommodate the multimodal and event-rich characteristics of the dataset.

### Implications for real-world industrial applications

The revelations of this research have direct consequences for intelligent manufacturing systems and Industry 4.0/5.0 environments. The feature hierarchy uncovered—where productivity metrics, learning-based signals, and security disturbances are the most prominent, offers a significant basis for actionable system monitoring. The hybrid models’ precision and robustness allow manufacturers to foresee efficiency declines, recognize the effects of cyber-physical disruptions, and provide interpretable, feature-aligned decisions to operators as a form of support.

In addition, the agreement between SHAP and ANOVA results guarantees that the model behaviors are always recognizable, thus giving support to the requirements coming from the regulatory and operational spheres in terms of explainability for cyber-physical production systems.

### Validation of SHAP-based explainability through feature perturbation

The SHAP-ranked features also align with expected operational behavior in intelligent manufacturing systems. Production Efficiency represents overall manufacturing throughput and process effectiveness, making its high contribution operationally meaningful for system-level efficiency prediction. Similarly, machine usage reflects resource utilization intensity and production workload conditions, which directly influence manufacturing stability and operational continuity. Q-value, which represents agent-learning quality and decision consistency, demonstrates a strong influence within the hybrid predictive framework, indicating the importance of adaptive learning behavior in multi-agent environments. In addition, Security Event contributes significantly to prediction outcomes due to its association with abnormal operational states and cyber-physical disturbances. The consistency between SHAP importance rankings and expected industrial behavior improves confidence in the practical validity of the explainability analysis.

To further validate the reliability of SHAP-based feature importance, a feature perturbation analysis was conducted using a One-at-a-Time (OAT) strategy. Controlled perturbations were independently applied to highly ranked SHAP features, including production efficiency, Q-value, and machine usage, while monitoring changes in predictive performance. The perturbation results indicate that features identified as highly influential by SHAP also produce the largest degradation in prediction quality when disturbed. In particular, perturbations applied to production efficiency and Q-value resulted in the most significant reductions in ${R}^{2}$ and corresponding increases in RMSE. This consistency between SHAP importance rankings and perturbation sensitivity supports the reliability of the explainability framework and confirms that the identified features are not merely statistical artifacts but represent stable predictive contributors within the hybrid learning architecture. The perturbation-driven degradation patterns are strongly aligned with SHAP feature rankings, thereby providing additional validation for the interpretability and stability of the explainability results.

### Limitations and practical considerations

Although the proposed models deliver high predictive performance, several practical limitations remain. First, optimization-enhanced hybrids can be computationally intensive during training, especially for large-scale real-time deployments. Second, the dataset, while richly multi-modal, still represents a controlled simulation scenario; real factories may introduce additional noise, shift, and uncertainty. Third, the reliance on tree-ensemble backbones means that extreme distributional drifts may still require periodic retraining to preserve predictive reliability. Finally, while explainability methods such as SHAP and ANOVA offer meaningful insights, they should always be interpreted alongside domain knowledge to avoid over-attribution or misinterpretation of complex industrial behaviors.

**Fig. 9:**
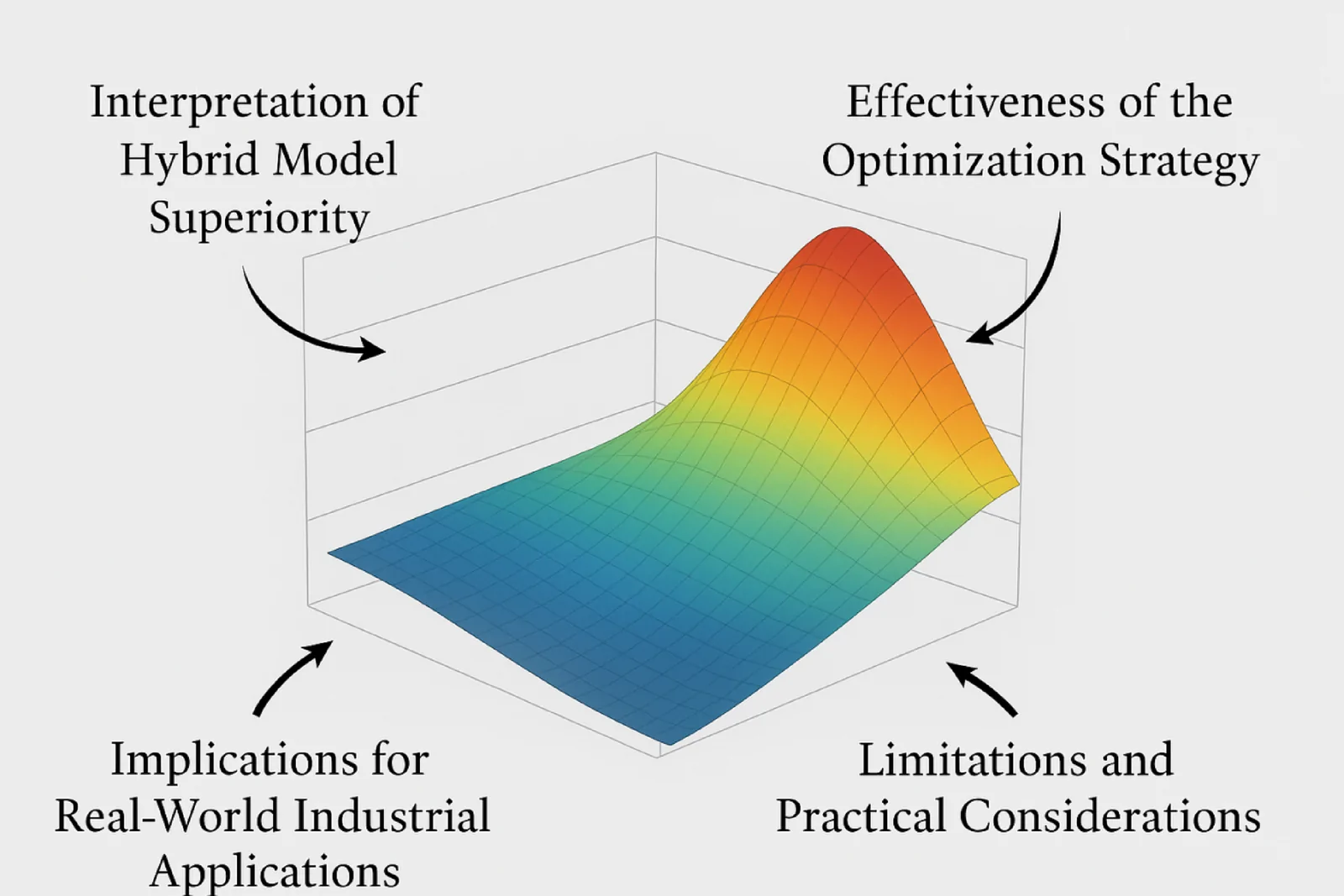
3D visualization of the discussion themes.

Displayed in Fig. 9 is a 3D performance surface visualizing the major insights derived from the research: the dominance of the hybrid model, the efficiency of the optimizer, the feasibility in the real world, and the restrictions of the practice. The increasing slope signifies enhanced model robustness, and the outlined areas function as an abridged version of each discussion component.

The proposed framework is intended as a deployment-ready decision-support architecture; however, its practical deployment in real industrial environments requires further validation using real-world operational datasets. While the proposed feature-centric decision-making framework demonstrates strong predictive performance and interpretability on the benchmark multi-agent manufacturing dataset, its evaluation is limited to simulated or publicly available industrial data. As such, direct external validation in real-world manufacturing environments has not been conducted. This may limit immediate generalizability to operational industrial systems that involve additional sources of noise, heterogeneous machine behavior, or real-time sensor variability. Nevertheless, the benchmark dataset used in this study is specifically designed to emulate complex multi-agent industrial conditions, including operational variability, system degradation, and cyber-physical interactions. Therefore, it provides a controlled and standardized environment for robust methodological validation and comparative analysis of predictive and optimization approaches. The proposed framework is modular and designed for deployment, which enables straightforward adaptation to real-world industrial datasets when they become available. Future work will focus on applying the framework to operational manufacturing IoT data, incorporating real-time streaming analytics, and validating performance across diverse industrial settings to strengthen external validity and practical applicability.

## Conclusion

This research presents a feature-driven hybrid predictive framework that comprehensively tackles the growing analytical complexity of intelligent multi-agent manufacturing systems. By strategically incorporating feature selection, dual-layer sensitivity analysis, and meta-heuristic optimization into the proposed pipeline, the authors achieve a synthesis of accuracy, interpretability, and operational relevance that is well-balanced. The use of Recursive Feature Elimination (RFE) is the main idea around which the framework revolves. This method reduces dimensionality without losing the semantic richness of multi-modal manufacturing data. As a result, the subsequent models will be able to focus on the most operationally meaningful variables. The authors supplement the feature selection with sensitivity analyses—statistical variance attribution by ANOVA and global model explainability by SHAP—which confirm feature effects and thus lead to a consistent ordering that is production efficiency, machine usage, Q-Value, and security event-dominated.

The congruence between the statistical and model-embedded insights constitutes the basis for a strong trust both in the modeling process and the resulting interpretations. The program optimization of the hybrid models through PDO and EEFO opens the way for them to outperform conventional tree-based learners. Their dynamic exploration–exploitation abilities allow them to effectively operate in nonlinear hyperparameter landscapes leading to quicker convergence, fewer errors, and better generalization of training, validation, and testing results. Among these, random forest and extra trees regressors enhanced by PDO-optimization along with the highest stability and accuracy are the most evident results of the optimizer’s power to capture complex multi-agent dynamics and event-driven fluctuations. The research results are also beyond sorts of technical outcomes, they are, for instance, significant implications for real-world smart manufacturing.

The models’ feature-centric architecture unveils to engineers and decision-makers the associated with higher contribution of operational behaviors, control adjustments, and cyber-physical disturbances on system efficiency. Such interpretability is at the core of predictive maintenance, production planning, and resilience management in Industry 4.0/5.0 scenarios. Moreover, the framework is aware of the existing limitations: computational costs during optimization, possible performance sensitivity to noise from the real world, and the need for periodic retraining due to changing operational conditions. In essence, this work moves forward the level of predictive intelligence in industrial systems through the introduction of a novel, explainable, and high-performing hybrid modeling paradigm. The integration of feature selection, sensitivity analysis, and meta-heuristic optimization into a single unified and deployable architecture in the study serves as a powerful strategic plan for cutting-edge analytics in intelligent multi-agent manufacturing environments.

## Data Availability

The data used in this study are available from the corresponding author upon reasonable request. No proprietary or confidential data were used.
